# The Discovery of RGH-706, a Highly Efficacious MCH1 Receptor Antagonist, for the Treatment of Obesity and Insatiable Hunger

**DOI:** 10.1021/acs.jmedchem.5c02708

**Published:** 2026-01-22

**Authors:** Gyula Beke, András Boros, György M. Keserű, Balázs Krámos, Krisztina Katalin Szalai, Anikó Gere, Mónika Vastag, Márta Thán, Balázs Varga, Ottilia Balázs, Sándor Farkas, Balázs Lendvai, István Greiner, János Éles

**Affiliations:** 58957 Gedeon Richter Plc., 19-21 Gyömrői út, Budapest 1103, Hungary

## Abstract

The discovery and characterization of a novel 2,3,4,5-tetrahydro-1*H*-[1,4]­diazepino­[1,7-*a*]­indole derivative MCH1 receptor antagonist (**37**) is disclosed. Starting from our previously investigated pyrazino­[1,2-*a*]­indole series and utilizing a scaffold hopping strategy, pyrimidine- and 1,4-diazepine-fused indole derivatives were designed and synthesized. Among these, only the prototype molecule containing the 2,3,4,5-tetrahydro-1*H*-[1,4]­diazepino­[1,7-*a*]­indole scaffold emerged as a chemically stable and potent MCHR1 antagonist. Previous SAR knowledge coupled with an ex vivo occupancy assay helped us to optimize this advanced lead to our candidate (**37**). The high MCHR1 potency and excellent receptor occupancy profile of **37** translated into statistically significant body weight loss after 14 days in a DIO mice study, supporting the potential use of this compound as a weight loss agent. Compound **37** (RGH-706) has successfully completed a phase I (SAD & MAD) clinical study in the indication of obesity, followed by an exploratory Phase II study in patients with Prader–Willi Syndrome (PWS).

## Graphical abstract

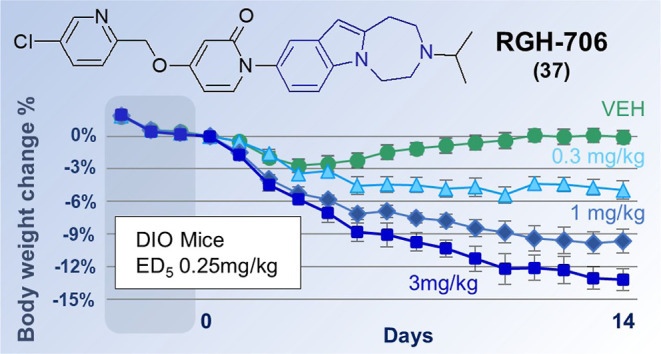

## Introduction

The obesity pandemic hits nearly all countries but paradoxically it is most prevalent in low- and medium-income economies. One billion of obese (BMI > 30 kg/m^2^) patients are predicted for the third decade of the century. Globally some 160 million years of healthy life were lost due to obesity and associated metabolic diseases in 2019. Recently the overall prevalence of obesity is 36% in the US and 30% in EU.^1^ Effective drugs have been available since 2012, but an unmet need is not fully met. The therapeutic breakthrough is limited by troublesome side effects and intolerance, which limit a patient’s adherence and a physician’s trust. The approval of glucagon-like peptide analogues and peptidomimetics is extremely effective in higher doses, but most of the patients do not tolerate them well (discontinuation reaches 70%), and the combination of the long lifetime and the risk of the severe adverse events put patients at high risk, which does not compensate the benefits in overweight patients.^2^ Accordingly, there is still room for a safe, effective, and better-tolerated drug in the metabolic field involving a CNS regulatory mechanism.

A melanin-concentrating hormone (MCH) as a neurotransmitter/neuromodulator attracted great biomedical interest as the only known hypothalamic peptide unequivocally and greatly contributing to weight gain and metabolic impairment in any preclinical settings.^3^ MCH is produced exclusively in the lateral hypothalamic (LH) and incertohypothalamic area and some minor extrahypothalamic cell groups. The pivotal importance of this localization is that LH area links homeostatic feeding and food reward pathways.^4^ MCH-erg axons radiate to all higher centers like ventral and dorsal striatum, prefrontal cortex, amygdala, ventral hippocampus, medullary vegetative nuclei, and the spinal cord. The cognate receptors for MCH are the GPCR MCHR1 (the only one in rodents), and its paralogue MCHR2 is also present in higher mammals and human. MCHR1 structure and signaling repertoire are well-known, while the role of MCHR2 is less studied. Preclinical studies with dogs and NHPs proved that MCHR1 is responsible for increased food intake and body weight gain, and MCHR2 does not compensate the effect of MCHR1 antagonists.^5^ The MCH-MCHR1 system has been also been implicated in a wide variety of other physiological functions and behaviors, including sleep pattern regulation, reward, anxiety, depression, and learning, but no MCHR1 antagonist targeting these indications has progressed to a clinical testing.^3,6,7^ The race to reach the obesity market started as early as 2004 with GSK’s candidate GW856464 and the compounds of four other companies (Amgen, Neurogen, Bristol Meyers Squibb, and AstraZeneca) followed and failed in Phase I clinical development (Figure 1).^7^ Only one compound, ALB-127158a (AMRI, Phase I completed in 2011)^8^ is probably still alive (Figure 1), following changes in the code (CSTI-100) and indications, ranging from obesity, inflammatory bowel disease, nonalcoholic fatty liver disease, hyperphagia in PWS to sleep disorders under the last name HBS-102, acquired by Harmony Biosciences.^9^ The scientific rationale for weight management with an MCHR1 antagonist is well established as MCH is one of the most potent central stimulators of feeding, and it regulates energy balance. However, the pivotal role of MCHR1 in syndromic obesity (PWS) has recently been revealed. The syndromic obesity driven by profound hyperphagia might arise from neurodevelopmental consequences of the deletion of the small nuclear C/D box RNA 116 (SNORD116) cluster.^10,11^ SNORD116 as a translational regulatory RNA has a major impact on prenatal development of hypothalamic orexinergic (ORX) neurons that are depleted to 40% in PWS mice. At the same time, the MCH-ergic cell population remains unaffected.^12,13^ MCH and ORX neurons exert antagonistic action on the energy balance.^14^ Developmental deficiency of ORX neurons leaves MCH activity unleashed, which in turn results in insatiable hunger in PWS.

**Figure 1:**
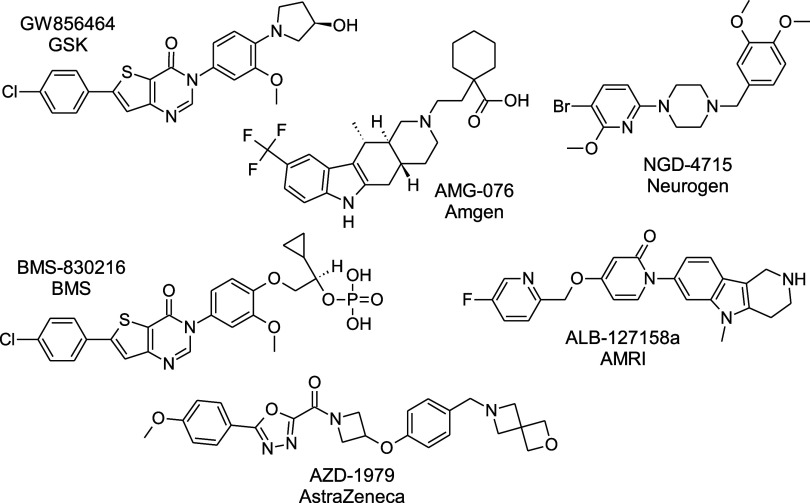
MCHR1 antagonists with clinical trials’ history.

In our previous paper^15^ we described the synthesis and structure–activity relationship of MCH1 receptor antagonists, incorporating 1,2,3,4-tetrahydropyrazino­[1,2-*a*]­indole (**1**) and 1,2,3,4-tetrahydro[1]­benzofuro­[3,2-*c*]­pyridine (**2**) scaffolds (Scheme 1), which had been identified by surveying in-house projects for tricyclic building blocks with basic amine functionality and an appropriate growing vector to be attached to the nitrogen of 4-(benzyloxy)­pyridin-2­(1*H*)-one (**6**) (Scheme 2).

**Scheme 1:**
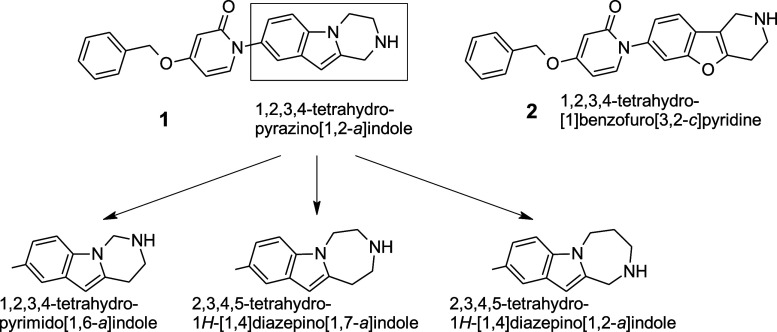
New Tricyclic Building Blocks Derived from the 1,2,3,4-Tetrahydropyrazino­[1,2-*a*]­indole Scaffold

**Scheme 2:**
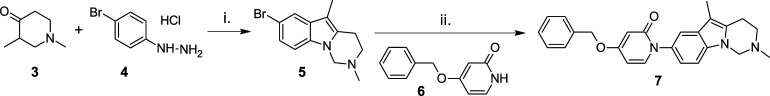
Synthesis of Target Molecule **7** Containing the 1,2,3,4-Tetrahydropyrimido­[1,6-*a*]­indole Scaffold^a^

a Reagents and conditions: (i) (1) EtOH, 1 h reflux; (2) HCl in EtOH (*w* = 28%), 70 min, reflux, 70%. (ii) CuI, Cs_2_CO_3_, *trans*-*N*,*N*′-dimethylcyclohexane-1,2-diamine, toluene, 110 °C o/n, 50%.

Although the in vitro profiles of the compounds optimized from **1** and **2** rendered them ideal tools for further pharmacological investigations, we wanted to find additional new tricyclic building blocks along the same lines. Accordingly, we devised three structures from the 1,2,3,4-tetrahydropyrazino­[1,2-*a*]­indole core as key scaffolds of prospective target molecules (Scheme 1). The first scaffold we designed was 1,2,3,4-tetrahydropyrimido­[1,6-*a*]­indole, in which the basic nitrogen of the template 1,2,3,4-tetrahydropyrazino­[1,2-*a*]­indole scaffold is shifted toward the indole nitrogen to form a formaldehyde aminal derivative. Next we planned to synthesize 2,3,4,5-tetrahydro-1*H*-[1,4]­diazepino­[1,7-*a*]­indole, in which the piperazine ring of the 1,2,3,4-tetrahydropyrazino­[1,2-*a*]­indole scaffold is expanded to contain an ethylenediamine unit. Finally we aimed to investigate 2,3,4,5-tetrahydro-1*H*-[1,4]­diazepino­[1,2-*a*]­indole, in which the basic nitrogen atom is further shifted away from the indole nitrogen in the previously mentioned scaffold.

## Results and Discussion

Representative examples (**7**, **16**, and **30**) containing the three scaffolds were synthesized as depicted in Schemes 2–4. In the synthesis of 1,2,3,4-tetrahydropyrimido­[1,6-*a*]­indole-derivative compound **7**, the key intermediate tricyclic scaffold **5** was synthesized in a Fischer cyclization reaction of the hydrazone intermediate obtained in the reaction of 1,3-dimethyl-4-piperidone (**3**) and 4-bromophenylhydrazine hydrochloride (**4**), based on a literature method.^16^ A copper­(I) iodide-catalyzed Ullmann-type C–N coupling of **5** with 4-benzyloxy-2­(1*H*)-pyridone (**6**) yielded target structure **7**.

**3:**
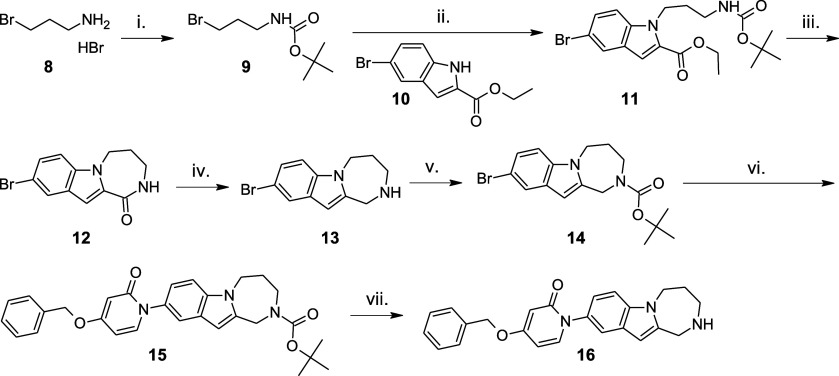
Synthesis of Target Molecule **16** Containing the 2,3,4,5-Tetrahydro-1*H*-[1,4]­diazepino­[1,2-*a*]­indole Scaffold^a^

a Reagents and conditions: (i) BOC_2_O, NaOH, H_2_O, DCM, rt 2.5 h, 96%. (ii) NaH 60%, KI, DMF, 0 °C–rt o/n, 74%. (iii) (1) TFA, DCM, rt 90 min; (2) K_2_CO_3_, MeOH, THF, rt 72 h, 67%. (iv) (1) BH_3_–Me_2_S 2.0 M in toluene, 70 °C 4 h; (2) HCl in water (6 N), EtOH, 80 °C 1 h; (3) DDQ, THF, rt 72 h. (v) BOC_2_O, TEA, DCM, rt o/n, 46% (2 steps, from **12** to **14**). (vi) **6**, CuI, K_2_CO_3_, 8-hydroxyquinoline, 3 Å MS, DMSO, 130 °C 46 h, 89%. (vii) HCl in EtOAc (*w* = 20%), MeOH, rt 2 h, 35%.

**Scheme 4:**
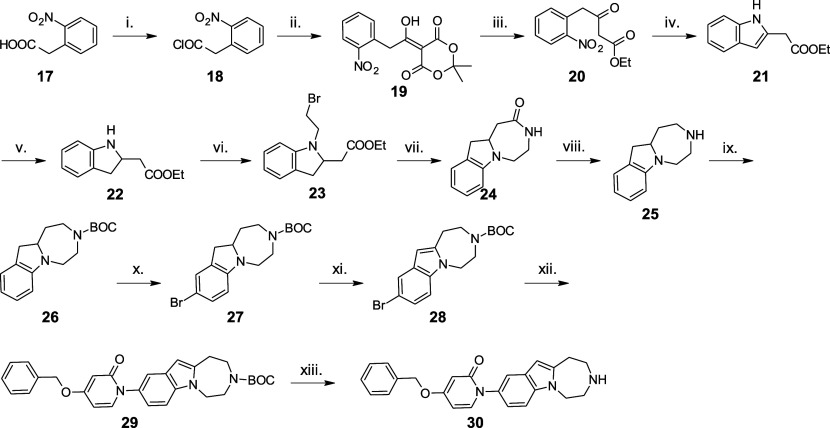
Synthesis of Target Molecule **30** Containing the 2,3,4,5-Tetrahydro-1*H*-[1,4]­diazepino­[1,7-*a*]­indole Scaffold^a^

a Reagents and conditions. (i) (COCl)_2_, DMF (cat.), DCM, rt 2 h. (ii) Meldrum’s acid, DIPEA, DCM, 0 °C–rt 2 h. (iii) EtOH, reflux 30 min, 82% (3 steps). (iv) H_2_ (1 atm), Pd–C, EtOH, 20 h, 25–40 °C, 99%. (v) NaCNBH_3_, AcOH, rt 90 min, 90%. (vi) BrCH_2_CH_2_Br, K_2_CO_3_, MeCN, reflux 96 h, 61%. (vii) NH_3_ in EtOH (*w* = 19%), EtOH, 4.3 bar, 50 °C 49 h, 95%. (viii) (1) BH_3_–Me_2_S in THF (2 M), THF, reflux 21 h; (2) HCl in water (6 N), EtOH reflux 1 h 77%. (ix) BOC_2_O, TEA, DCM, rt 3.5 h, 94%. (x) NBS, MeCN, rt 2.5 h, 80%. (xi) DDQ, THF, 0 °C 45 min, 82%. (xii) **6**, CuI, Cs_2_CO_3_, *trans*-*N*,*N*′-dimethylcyclohexane-1,2-diamine, toluene, 110 °C o/n, 84%. (xiii) HCl in EtOAc, EtOAc, rt 2 h, quantitative.

In vitro screening revealed that compound **7** (tested as maleic acid salt) had high MCH1 receptor antagonist functional activity (IC_50_ 14.7 nM) as well as low microsomal clearance (Cl_int_ mouse/human 3.3/10 μL/min/mg protein), but the presence of the geminal diamine moiety warranted chemical stability testing. Although the compound proved to be stable in DMSO and SGF (pH 1.2), it decomposed with the loss of formaldehyde to varying extents in other matrices (pH 2.8; SIF (pH 6.8); PBS (pH 7.4), and pH 9.0) to the 2-[2-(methylamino)­ethyl]-1*H*-indol-5-yl-derivative. Consequently, further activity with this scaffold was terminated.

The 2,3,4,5-tetrahydro-1*H*-[1,4]­diazepino­[1,2-*a*]­indole scaffold of **16** was constructed from **11** prepared in the N-alkylation reaction of ethyl 5-bromoindole-2-carboxylate (**10**) with BOC-protected 3-bromopropylamine (**9**) and sodium hydride as the base. After the removal of the BOC-protecting group of **11**, treatment with potassium carbonate in methanol initiated the closure of the diazepinon ring to obtain **12**. Its reaction with the borane dimethyl sulfide complex in toluene provided **13** along with the side product indoline-derivative, which was reoxidized to the target intermediate with DDQ in the mixture. The so obtained basic secondary amine **13** was BOC-protected (**14**) and copper-catalyzed C–N coupling with 4-benzyloxy-2­(1*H*)-pyridone (**6**) yielded **15**, which was deprotected using HCl in ethyl acetate to provide the desired compound **16**.

The synthesis of compound **30** centered on the copper-catalyzed C–N coupling of *tert*-butyl 9-bromo-1,2,4,5-tetrahydro-3*H*-[1,4]­diazepino­[1,7-*a*]­indole-3-carboxylate (**28**) with 4-benzyloxy-2­(1*H*)-pyridone (**6**) to obtain **29**, which was deprotected using HCl in ethyl acetate to provide **30** (Scheme 4).^17^ In the synthesis of the bromo-derivative coupling partner **28** we have previously described the preparation of intermediate **24** from 2-nitrophenylacetic acid (**17**) in seven steps, where flow chemistry techniques were also applied to improve some steps of the discovery chemistry route.^18^ Next, by applying literature procedures,^19,20^ lactam **24** was reduced with the borane–dimethyl sulfide complex to provide the basic indoline (**25**), which was *N*-BOC-protected (**26**) and regioselectively brominated with NBS to provide indoline **27**. Subsequently, the oxidation of **27** with DDQ provided the targeted compound **28**.^17^

Although structures **16** and **30** are closely related to each other, to our great surprise the 2,3,4,5-tetrahydro-1*H*-[1,4]­diazepino­[1,2-*a*]­indole derivative (**16**) is functionally much less active antagonist on the MCH1 receptor than its isomer **30** containing the 1,2,3,4-tetrahydropyrazino­[1,7-*a*]­indole scaffold (Table 1); consequently no more derivatives of compound **16** were prepared. Characterization of **30** revealed suboptimal mice and human metabolic stability. Notwithstanding the latter results, brain penetration and target engagement of **30** were estimated by ex vivo brain MCH1 receptor occupancy measurements using mouse striatum homogenate and the radioligand [^3^H]­SNAP7941.^21^ Orally administered **30** inhibited the binding of the radioligand with an ED_50_ of 9 mg/kg and an *E*_max_ of 84% at 30 mg/kg, clearly showing the presence of the compound in the target organ, comparable to the data of ip-administered compound **1**.

**Table 1:** In Vitro and Ex Vivo Data of Compounds **16**, **30**, Compared to **1**

		Cl_int_ ^a^ (μL/min/mg protein)		ex vivo receptor occupancy in mice		
no.	MCHR1 IC_50_ (nM)	mouse	human	ED_50_ (mg/kg)	*E*_max_ (%) (30 mg/kg)	route of adm.
**1**	39.9	13	22	4	86 (2 h)	ip
**16** ^b^	1038	5.2	1.4	-	-	-
**30** ^c^	17.7	67	14	9	84 (4 h)	po

a C57BL/6 mouse and human intrinsic clearance values in microsomes.

b Monohydrochloride salt.

c Maleic acid salt.

In accordance with strategic decisions related to the forthcoming conclusion of the project, we initially opted to prepare only the two most promising derivatives of **30**. Structure–property relationships obtained when working with MCH1 receptor antagonists derived from 1,2,3,4-tetrahydropyrazino­[1,2-*a*]­indole (**1**) and 1,2,3,4-tetrahydro[1]­benzofuro­[3,2-*c*]­pyridine (**2**) scaffolds indicated that the replacement of the phenyl ring of the benzyl moiety by a 5-chloropyridine-2-yl ring generally improved lipophilicity-related parameters, e.g., metabolic stability and hERG affinity.^15^ Furthermore, the introduction of an alkyl-substituent to the secondary basic amine nitrogen seemed to be a promising approach to improve the brain penetration of **30**, by means of removing the HBD functionality and masking some of the polarity, with the consequence of increasing lipophilicity. For this reason, the isopropyl group was chosen, representing an optimum bulkiness and lipophilicity.

The syntheses of the targeted compounds **36** and **37** are shown in Scheme 5.^17^ Substitution of the nitro group in 4-nitropyridine-*N*-oxide (**31**) with (5-chloro-2-pyridinyl)­methanol (**32**) followed by the acetic anhydride-mediated rearrangement of the corresponding arylmethoxypyridine-*N*-oxide **33** yielded 4-[(5-chloropyridin-2-yl)­methoxy]­pyridin-2­(1*H*)-one as described in our previous publication.^15^ The subsequent copper-catalyzed C–N coupling with **28** and BOC-deprotection using HCl in ethyl acetate provided **36**. The latter was alkylated at the secondary nitrogen by isopropyl iodide in acetonitrile and with potassium carbonate as the base to obtain **37**.

**Scheme 5:**
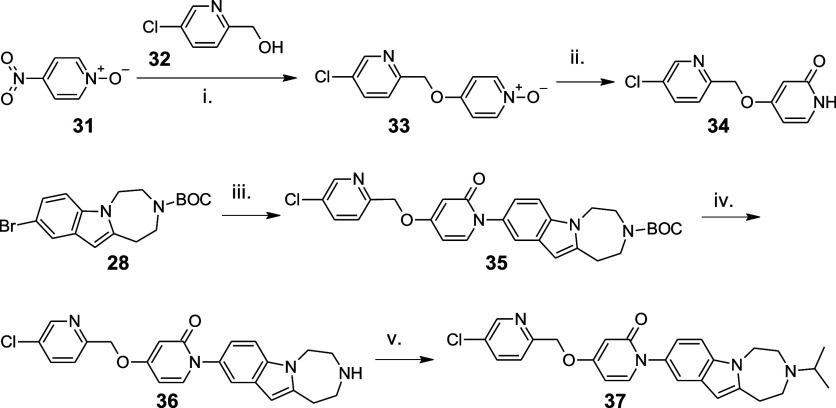
Synthesis of Target Molecules **36** and **37** ^a^

a Reagents and conditions: (i) DCM, BTEAC, NaOH (aq), RT, 12 h, 42%. (ii) Ac_2_O, reflux, 4 h, 38%. (iii) **34**, CuI, Cs_2_CO_3_, *trans*-*N*,*N*′-dimethylcyclohexane-1,2-diamine, toluene, o/n 100 °C, 66%. (iv) HCl in EtOAc (*w* = 20%), MeOH, rt o/n, quantitative. (v) 2-Iodopropane, K_2_CO_3_, MeCN, reflux 45 h, 64%.

As can be seen in Table 2, the objectives of improved metabolic stability and target organ exposure could be achieved by the adopted strategy, without compromising MCH1 receptor functional activity. The replacement of the phenyl ring of the benzyl moiety in **30** by the 5-chloropyridine-2-yl ring provided **36** with higher human metabolic stability and somewhat improved median effective dose of 3.8 mg/kg (po) in the ex vivo brain MCH1 receptor occupancy measurement in mice with a *E*_max_ of 85% at 30 mg/kg within the 4 h time frame. The PDR value obtained from the VB-Caco-2 penetration assay^22,23^ indicated that **36** was a P-glycoprotein substrate, which was favorably altered by the introduction of the isopropyl group to the secondary basic amine nitrogen (**37**), while preserving in human and improving in mice the metabolic stability and significantly decreasing the ED_50_ value (0.4 mg/kg) of ex vivo receptor occupancy in mice. To our delight, the maximum attainable receptor occupancy of **37** proved to be 99%, at as low as 3 mg/kg, at 4 h postdose.

**Table 2:**
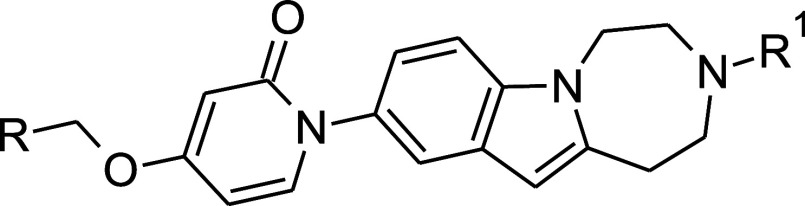
In Vitro and Ex Vivo Data of Compounds **36** and **37**, Compared to Compound **30**

					Cl_int_ ^b^ (μL/min/mg protein)		penetration VB-Caco-2		ex vivo receptor occupancy in mice, po, 4 h postdose	
no.	R	R^1^	*c* log *P*/log *D* ^a^	MCHR1 IC_50_ (nM)	m	h	*P*_app in_ ^c^	PDR	ED_50_ (mg/kg)	*E*_max_ (%)
**30** ^d^	Ph	H	3/2.42	17.7	67	14	-	-	9	84 (30 mg/kg)
**36** ^e^	5-Cl-Pyr-2-yl-	H	2.5/1.11	9.8	13	11	9.2	4.5	3.8	85 (30 mg/kg)
**37** ^d^	5-Cl-Pyr-2-yl-	^*i*^Pr	3.7/3.15	6.2	4.2	7.8	23	0.9	0.4	99 (3 mg/kg)

a Determined using potentiometric titration (T3-Sirius Instrument, Sirius Analytical Inc.).

b C57BL/6 mouse and human intrinsic clearance values in microsomes.

c *P*_app in_ (×10^–6^ cm/s).

d Maleic acid salt.

e Dihydrochloride salt.

Brain penetration of **37** was further quantified by a single oral dose pharmacokinetic measurement in mice (Table 3, Figure 2). After 10 mg/kg po administration, high maximal concentrations in plasma and brain were reached at 0.5 h, while the terminal half-life proved to be 2.71 h in plasma. For the assessment of the extent of CNS distribution the brain-to-plasma ratio was calculated as AUC_0–24_ brain/plasma and it was decently high (2.06), while the unbound brain-to-plasma ratio, calculated from the same brain and plasma AUC values using the fraction unbound of brain and plasma (*f*_b,u_ 0.009, *f*_p,u_ 0.0235), was somewhat lower (0.79).

**Table 3:** Plasma and Brain PK Parameters of **37** (Maleic Acid Salt) in Mice at 10 mg/kg po^a^

	AUC_0–24_ (μM h)	*C*_max_ (μM)	*T*_max_ (h)	*T*_1/2_ (h)	B/P	*f*_u_	B/P_(u)_
plasma	18.0	3.8	0.5	2.71	2.06	0.0235	0.79
brain	37.2	6.6	0.5	NC		0.009	

a NC: not calculable.

**Figure 2:**
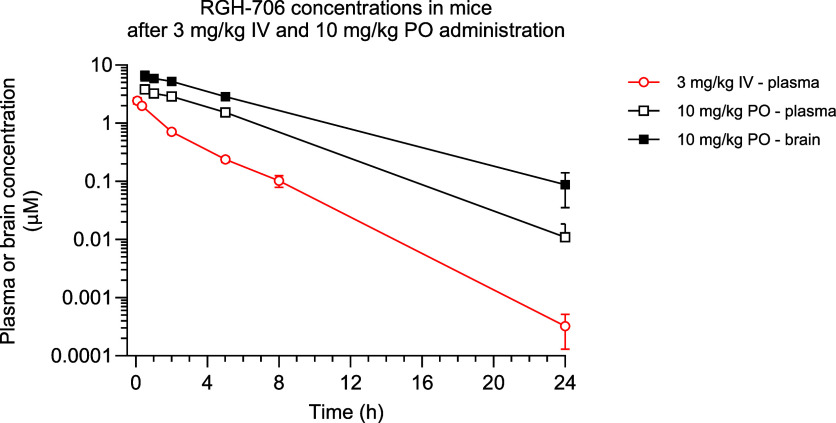
Pharmacokinetic curves of 37 (maleic acid salt) in mice at 3 mg/kg iv and 10 mg/kg po.

These results qualified compound **37** to be subjected to a body weight-lowering assay in a 14 day diet-induced obesity (DIO) model in mice. Since in the conditioned taste aversion (CTA) assay the maximum aversion-free oral dose proved to be 3 mg/kg in lean CD-1 mice, compound **37** was administered to obese C57BL/6 mice in 0.3, 1, and 3 mg/kg, twice daily. At the end of the 14 days treatment, control-subtracted body weight loss was calculated and analyzed using one-way ANOVA and Tukey’s post hoc tests. It was shown that compound **37** induced dose-dependent, though not dose-proportional, weight loss in all doses (BWL = 13.1% at 3 mg/kg b.i.d.) (Figure 3). We could also measure a sustained decrease in daily food intake (10–30%), which likely drives the weight reduction observed. Linear regression on the control subtracted body weight loss data provided an ED_5_ value of 0.25 mg/kg. In a 28 day DIO study in mice (3 mg/kg, b.i.d.), there was no statistically significant difference in body weight loss at day 28 compared to the 14 day study, showing sustained effect and no habituation to treatment.

**Figure 3:**
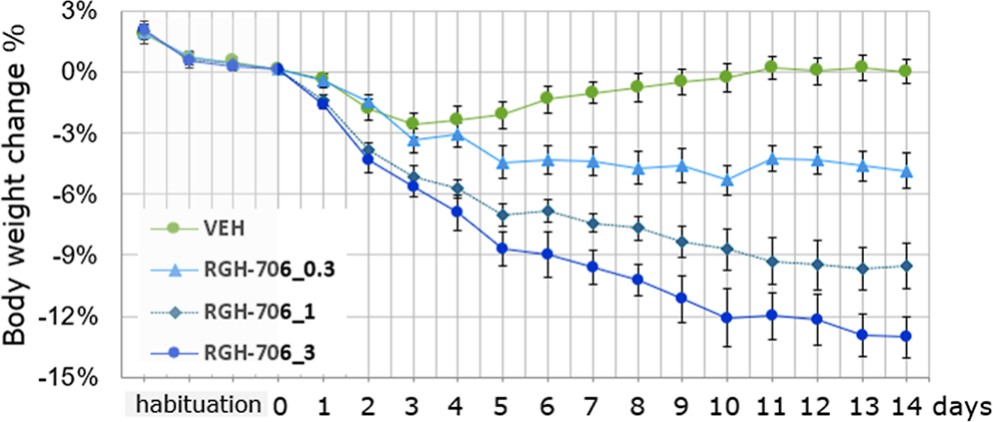
Dose–response curve of compound **37** (maleic acid salt; RGH-706) in DIO mice on control-subtracted body weight loss (%).

In this in vivo experiment plasma and brain concentrations of **37** were also measured 1 h after the first treatment of the 14th day (*C*_max_) and 18 h after the second treatment of the 13th day (*C*_min_). As shown in Table 4, the increase of plasma and brain concentrations was supraproportional in the dose range of 0.3–3 mg/kg. Based on these data, the minimal effective dose (MED, 0.3 mg/kg po, b.i.d.) was associated with 24% receptor occupancy and 0.37 *C*_u brain_/IC_50_ potency ratio, as the lower threshold required for minimal efficacy, while the maximal effective dose was associated with 99% receptor occupancy and 5.78 *C*_u brain_/IC_50_ potency ratio, where efficacy saturates.

**Table 4:** Plasma and Brain Concentrations of **37** (Maleic Acid Salt) in DIO Mice^a^

	plasma concentration (ng/mL)/(μM)		brain concentration^b^ (ng/g)/(μM)	
dose (mg/kg)	*C*_max_	*C*_min_	*C*_max_	*C*_min_
0.3	80.9 (10.5)/0.175	3.47 (28.5)/0.007	119 (9.9)/0.258	5.87 (22.6)/0.001
1	307 (13.7)/0.664	23.8 (31.6)/0.051	475 (5.7)/1.010	40.1 (17.7)/0.086
3	1160 (14.8)/2.51	174 (7.0)/0.376	1839 (19.2)/3.010	269 (18.3)/0.582

a *C*_max_ and *C*_min_ were measured at 1 h after the 1st treatment of the 14th day and 18 h after the 2nd treatment of the 13th day, respectively. Variability of observed concentrations (CV %) are indicated in parentheses.

b With the assumption of 1 mg/mL brain homogeneity density.

Considering the general structural features of MCHR antagonists (lipophilic groups linked to an aliphatic basic amine moiety), it is essential to screen against the K_v_11.1 (hERG) potassium channel.^24^ The hERG IC_50_ value of **37** was determined using the whole-cell patch-clamp-based assay on hERG channels at near physiological temperature (36 ± 1 °C) for potential long-QT and TdP risk assessment. The concentration response (inhibition % of hERG tail current amplitude) curve was generated based on actual concentrations, and the IC_50_ value was calculated to be 6.44 μM (Hill coefficient: 1.14) that was satisfactory for a preclinical development candidate, with a selectivity index (defined as a ratio between hERG and MCHR1 inhibitory potencies expressed as IC_50_) over 1000.

As compound **37** showed excellent in vitro and in vivo profiles, it was selected for an extensive characterization (Table 5).

**Table 5:** Detailed Profile of **37**

physicochemical properties	MW = 462.18
	experimental log *P* = 4.11, log *D* = 3.15 (pH = 7.4)
	experimental p*K*_a_ (pyridine) = 1.34, p*K*_a_ (aliphatic N) = 8.41
	thermodynamic solubility = 9.9 μM (pH = 7.4), 117 μM (pH = 6.5), 7699.3 μM (pH = 5.0)
in vitro pharmacology	hMCHR_1_ IC_50_ = 6.2 nM
	hMCHR_2_: no antagonistic activity
	off target activities^a^ (% at 10 μM/IC_50_ [μM]): hH_3_R 97/0.290; hD_3_R 85/no effect; hM_1_ 88/3.46; hM_2_ 84/3.85; hM_3_ 51/9.55; hM_4_ 71/6.89; rM (nonselective, central) 95/1.5; h5-HT_2C_ 55/12.3
ADME profile	microsomal stability: Cl_int_ (μL/min/mg protein) = 7.8 (human), 4.2 (mouse), 9.6 (rat), 11.3 (dog)
	permeability (VB Caco-2): *P*_app in_ 23 × 10^–6^ cm/s, PDR = 0.9 at 10 μM
	plasma protein binding (*f*_u_) = 0.0515 (human), 0.0235 (mouse), 0.0177 (rat), 0.0321 (dog)
	brain tissue binding, mouse (*f*_u_) = 0.009
	CYP450 inhibition: IC_50_ > 25 μM for 1A2, 2B6, 2C8, 2C9, 2C19, 2D6, 3A4/5
PK	mouse PK 3 mg/kg iv/10 mg/kg po: F % = 100%; B/P = 2.21 ♂
	mouse PK 10 mg/kg po: plasma *C*_max_: 3.8 μM, *T*_max_: 0.5 h, *T*_1/2_: 2.71 h, B/P = 2.06 ♂
	rat PK 1 mg/kg iv/3 mg/kg po: F % = 47.6%; B/P = 2.13 ♂
	dog PK 1 mg/kg iv/10 mg/kg po: F % = 46.8% (♂)/(♀)
in vivo pharmacology	mouse 14D-DIO: ED_5_ ^b^= 0.25 mg/kg po
	MED = 0.3 mg/kg po (free plasma conc. at 1.5 h postdose = 4.11 nM)
CNS safety	Irwin in rats: NOAEL ≥ 14.29 μM (100 mg/kg), po, PLR^c^≥ 81
CV safety	hERG IC_50_ = 6.44 μM
	dog CV: NOAEL ≥ 3.81 μM (10 mg/kg), po, PLR^c^≥ 22
toxicology	Ames: clean up to 5000 μg/plate (±S-9)
	4 week oral rat tox: NOAEL ≥ 5.84 μM (30 mg/kg/day)

a The Eurofins Panlabs, Inc. LeadProfiling Screen (total number of assays: 91). M = muscarinic.

b Dose for 5% weight loss.

c The plasma level ratio (PLR) was calculated as the ratio of the safety NOAEL (Irwin 14.29 μM; dog CV 3.81 μM) and the plasma level of the mouse 14D-DIO ED_5_ (0.175 μM).

Compound **37** exhibited acceptable physicochemical properties. It showed high subtype selectivity against hMCH_2_ receptors. The most prominent off-target activity proved to be hH_3_ receptor antagonism (IC_50_ = 290 nM), but no pharmacological effect could be associated with this liability. Moreover, the compound produced >50% inhibitory effect on hD_3_R, hM_1–4_, rM (nonselective, central) and h5-HT_2C_ receptors with at least 240 times selectivity with respect to the MCH_1_ receptor in the functional assay (Table 5). Compound **37** is a high microsomal metabolic stability compound and does not inhibit CYP450 enzymes significantly. VB Caco-2 data showed a high permeability without efflux liability. The level of plasma protein binding was consistently high across the studied species. Oral bioavailability was moderate in rats and dogs and high in mouse. The compound produced significant body weight loss in a 14 day diet-induced obesity (DIO) model in mice. Compound **37** was free of CNS side effects in the rat Irwin test and did not alter CV parameters significantly in dogs in the applied doses. A 4 week oral toxicity study in rats did not reveal any adverse effects up to the highest dosage of 100 mg/kg/day. Compound **37** did not show any indication of genotoxicity in vitro (Table 5).

In order to glean some insight into the significant activity difference between the compounds containing the 2,3,4,5-tetrahydro-1*H*-[1,4]­diazepino­[1,2-*a*]­indole (**16**) and 2,3,4,5-tetrahydro-1*H*-[1,4]­diazepino­[1,7-*a*]­indole (**30**, as well as scaffolds **36**, **37**) scaffolds, retrospective modeling studies have been carried out. For this, we used the recently published experimental structure of the MCHR1 receptor (PDB id: 8WSS)^25^ rather than previous homology models.^26–30^ The possible binding modes of compound **37** were explored using induced-fit docking (IFD), which can also take the binding site flexibility into account to a limited extent.^31^ Diverse binding modes were obtained in accordance with the quite large binding site between the transmembrane helices where MCH also binds, but in most of top scoring poses the salt bridge formed with Asp-192^3.32^ residue appeared to be an important interaction, consistent with the literature,^26,28–30,32^ and the orientation of the binding modes was also similar (see Figure S1A in the Supporting Information).

From the IFD results two possible binding poses were selected that primarily differed in the orientation of the tricyclic headgroup. The structures were refined by using molecular dynamics (MD) simulations. Of the two binding modes, only one appeared stable (pose 1); in the other case the ligand significantly deviated from its initial position (see Figure S1C,D in the Supporting Information). Thus, only the model having compound **37** in pose 1 was examined further. The refined binding mode and the protein–ligand interactions are summarized in Figure 4A–C and in Table S1 in the Supporting Information. Beside the crucial salt bridge formed with the Asp-192^3.32^, the tricyclic headgroup can form further important interactions with the aromatic side chain of Tyr-341^6.51^ and Tyr-370^7.43^. In the MD simulation, the carbonyl of the ligand served as an important acceptor site interacting with Thr-278^5.39^, Gln-281^5.42^, and Gln-345^6.55^ and the aromatic tail was stabilized in a lipophilic pocket formed by Leu-267, Leu-274^5.35^, and Phe-277^5.38^.

**Figure 4:**
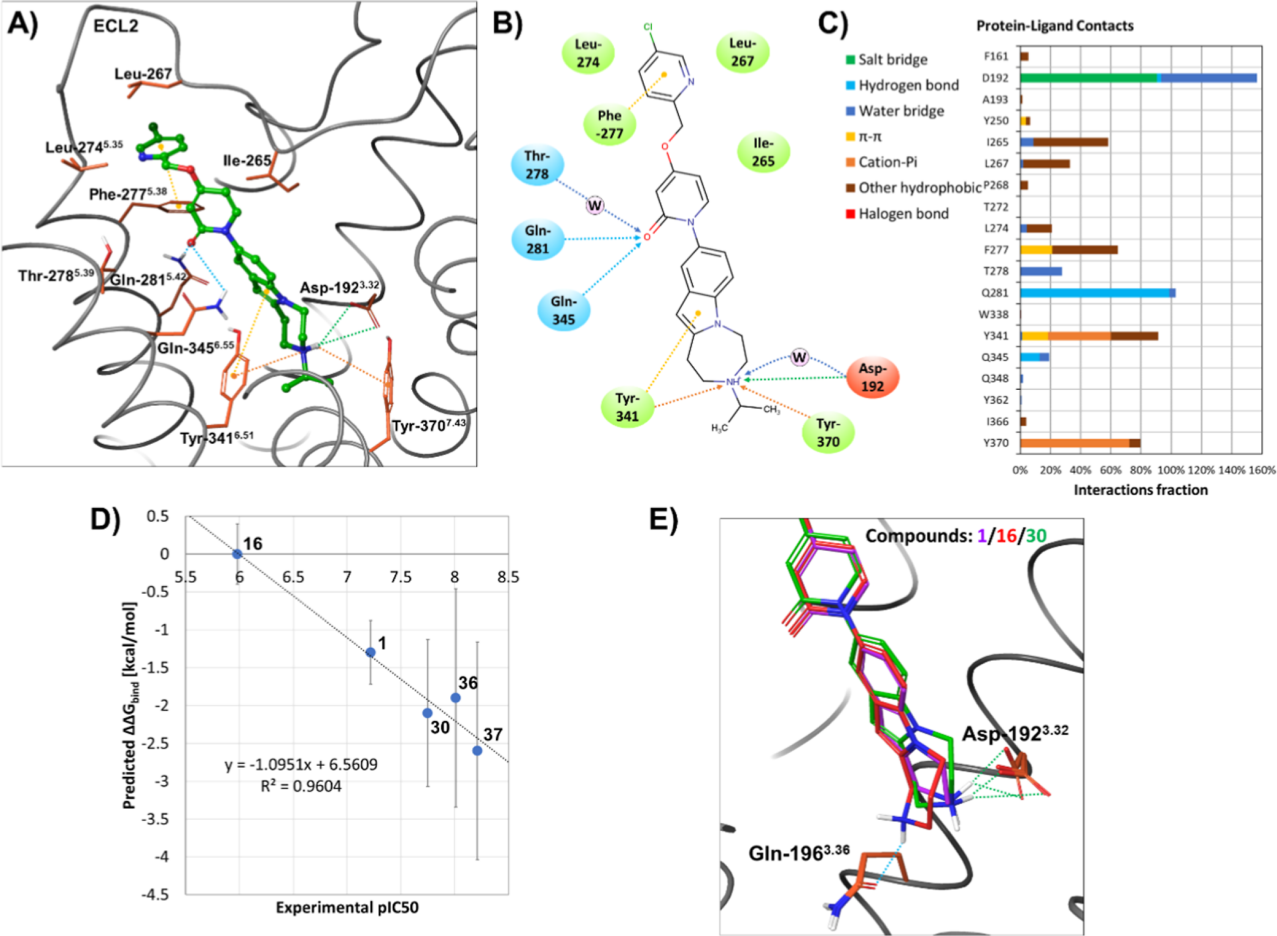
Summary of modeling results: (A) Molecular Dynamics-refined binding pose of compound **37**; (B) 2D representation of main specific protein–ligand interactions; (C) ligand–residue interaction statistics by counting different types of interactions per frame of the MD simulation. The range of 100 to 200 ns was analyzed. It should be noted that a single residue can form more than one counted interaction with the ligand at the same time, so the sum can be higher than 100%; (D) predicted relative binding free energies determined by FEP calculations versus the experimental pIC_50_ values; (E) differences in the position of the ionic center of the ligands, which affects the protein–ligand interactions (for initial geometries for FEP calculations, see the Supporting Information for 3D models).

The binding mode was further validated using free energy perturbation (FEP) calculations.^33–35^ Although FEP calculations are a technique for predicting relative binding affinities, the calculated affinity trend correlated well with the experimentally obtained functional IC_50_ data, as shown in Figure 4D. These results suggest that the difference in measured biological activities can be attributed to different binding affinities and confirm the correctness of the assumed binding mode of the ligands. Presumably the interaction between the basic amine group and the protein, primarily the salt-bridge-forming Asp-192 residue, plays a key role in the activity trend since compounds **1**, **16,** and **30** differ only in the aliphatic portion of their tricyclic headgroup. Figure 4E illustrates that the position and orientation of the amine function of compound **16** deviated the most from that of compound **1** and **30** in the starting structures used for the FEP calculations. During these calculations, multiple accessible conformational states of the headgroups were also considered thanks to the high-level sampling algorithm. Although the salt bridge could also be formed in the case of compound **16** during these simulations, this was only possible through a conformational flip of the seven-membered ring somewhat affecting the other interaction components, which could ultimately lead to a higher calculated relative binding free energy, in agreement with the experimental data. A more detailed analysis of protein–ligand interactions is provided in the Supporting Information (Figure S2).

## Conclusions

In summary, pyrimidine- and diazepine-anellated indole derivatives were synthesized and tested for MCH1 receptor activity. Among them, 2,3,4,5-tetrahydro-1*H*-[1,4]­diazepino­[1,7-*a*]­indole derivatives emerged as chemically stable and active receptor antagonists. Brain penetration and target engagement were estimated by ex vivo brain MCH1 receptor occupancy measurements in mice. Appropriately chosen substitution pattern of compound **37** resulted in an excellent receptor occupancy profile that translated to statistically significant body weight loss after 14 days in a DIO mice study, supporting the potential use of this compound as a weight loss agent. Compound **37** (RGH-706) has progressed to clinical development with the indication of obesity (Phase I, SAD, and MAD) and subsequently for the therapy of Prader–Willi syndrome (exploratory Phase II study). The Phase I studies were successful as RGH-706 was well tolerated, no serious adverse event appeared, and sign of efficacy was detected. The exploratory Phase II clinical study has been completed, and RGH-706 was safe, well-tolerated, and had shown a statistically significant effect on body weight in Prader–Willi syndrome patients (KITE-PWS, NCT05322096, EMA CTSI 2025).

## Experimental Section

### General Experimental Methods

Reactions were carried out under an inert gas (nitrogen or argon) when indicated. Commercially available reagents were used without further purification. Solvents and gases were dried according to the standard procedures. Organic solvents were evaporated under reduced pressure using a rotary evaporator.

All compounds are >95% pure by HPLC analysis.

The purity of final compounds was verified using HPLC-DAD analysis (Method 1), carried out on an Agilent 1100 series LC chromatographic system equipped with an autosampler, thermostated column compartment, quaternary pump, and diode array detector. Data were acquired using Agilent Chemstation software. The chromatographic column used is a Chromolith Performance RP-18e (150 mm × 3 mm), and the column temperature was set at 40 °C. The mobile phase consisted of 0.1% (v/v) trifluoroacetic acid in water/acetonitrile (95/5, v/v) (eluent A); and 0.1% (v/v) trifluoroacetic acid in water/acetonitrile (5/95, v/v) (eluent B). The gradient program was as follows: 0 min—0% B, 9.7 min—90% B, 12.5 min—90% B. The detection wavelength, flow rate, and injection volume were set at 220 nm, 1.7 mL/min, and 1.5 μL, respectively. The sample was dissolved in eluent B.

The identity and purity assay of all compounds (synthetic intermediates and final compounds) was performed by one of the next HPLC-MS methods:

Method 2: performed on an Agilent 1200 model HPLC system, consisting of a degasser, autosampler, binary pump, and column oven. The chromatographic system was coupled to an Agilent 6120 Single Quadrupole mass spectrometer equipped with an electrospray ionization source operated in positive ion mode. As dry gas, nitrogen with a gas flow of 12 L/min (350 °C) was used, the nebulizer was adjusted to 35 psig, and mass spectra were registered in the full-scan mode (*m*/*z*, 600–2000). Analysis was carried out at 45 °C on a Kinetex EVO C18 column (50 × 3.0 mm, 2.6 μm) (Phenomenex), with a mobile phase flow rate of 1.45 mL/min. The composition of eluent A was 0.1% (v/v) TFA in water and eluent B was 0.1% (v/v) TFA in acetonitrile. A linear gradient of 2–100% B was applied at a range of 0.0–4.9 min and then 100% B at 4.9–6.0 min.

Method 3: performed on a Shimadzu Prominence chromatographic system equipped with an autosampler, thermostated column compartment, degasser, binary pump, diode array detector, and coupled with a Shimadzu LCMS-2020 single quadrupole mass spectrometer. Data were acquired using LabSolutions software. The chromatographic column used was a Cortecs C18+ (50 mm × 3 mm; 2.7 μm) produced by Waters, and the oven temperature was set at 40 °C. The mobile phase consisted of 0.1% (v/v) formic acid in water as eluent A; and 0.1% (v/v) formic acid in water/acetonitrile (5/95, v/v) as eluent B. The gradient program was as follows: 0 min—5% B, 4.5 min—100% B, 6.6 min—100% B. The analysis was performed with a 1.25 mL/min flow rate. The detection wavelength was set at 240 nm with a 25 nm bandwidth. The injection volume was set at 1.5 μL and the sample was dissolved in acetonitrile. The MS detection was performed in an ESI positive mode. The ion source parameters were set as follows: 280 and 250 °C for DL and Heat Block temperature, respectively, 1.5 L/min for nebulizing gas flow, 12 L/min for drying gas flow, and 4.5 kV for the Interface Voltage. Nitrogen was used as a drying gas. The mass spectra were registered in scan mode (*m*/*z*, 121–900) with 790 u/s scan speed.

Method 4: performed on a Waters Aquity ARC chromatographic system equipped with an autosampler, thermostated column compartment, degasser, binary pump, and diode array detector, and coupled with a Waters QDa single quadrupole mass spectrometer. Data were acquired by using Empower software. The chromatographic column used was a Cortecs C18+ (50 × 3 mm; 2.7 μm) produced by Waters, the oven temperature was set at 40 °C. The mobile phase consisted of 0.1% (v/v) formic acid in water as eluent A; and 0.1% (v/v) formic acid in water/acetonitrile (5/95, v/v) as eluent B. The gradient program was as follows: 0 min—5% B, 4.5 min—100% B, 6.6 min—100% B. The analysis was performed with a 1.25 mL/min flow rate. The detection wavelength was set at 220 nm. The injection volume was set at 2 μL and the sample was dissolved in acetonitrile. The MS detection was performed in ESI positive mode. The ion source parameters were set as follows: 600 °C for probe temperature, 15 V for cone voltage, and 0.8 kV for capillary voltage. Nitrogen was used as drying gas. The mass spectra were registered in scan mode (*m*/*z*, 100–900) with 4.8 point/s scan speed.

Method 5: performed similar to Method 2, except that the analysis was carried out at 40 °C on a Kinetex EVO XB column (50 × 2.1 mm, 2.6 μm) (Phenomenex), with a mobile phase flow rate of 0.75 mL/min. The composition of eluent A was 0.1% (v/v) TFA in water and eluent B was 0.1% (v/v) TFA in water/acetonitrile (5/95, v/v). The gradient program was as follows: 0 min 0% B, 4 min 100% B, 7 min 100% B and 7.01 min 0% B.

All NMR samples were prepared in DMSO-*d*_6_. ^1^H, ^13^C, COSY, TOCSY, NOESY, HSQC, and HMBC spectra were recorded in standard 5 mm tubes at 298 K on a Bruker Avance III HDX system operating at 800 MHz, equipped with a TCI 800SA H&F/C/N-D-05 Z cryoprobe or on a Bruker Avance III HDX system operating at 500 MHz, equipped with a TCI 500S2 H-C/N-D-05 Z ET cryoprobe or on a Bruker Avance III HDX system operating at 400 MHz, equipped with a CPP BBO 400S1 BB-H&F-05 Z ET probe. Chemical shifts are referenced to TMS (0.00 ppm). All pulse sequences were applied by using the standard spectrometer software package (Topspin 3.5pl7).

Electrospray high-resolution MS measurements (ESI-HRMS) were performed on a Thermo Velos Pro Orbitrap Elite Hybrid Mass spectrometer (Thermo Fisher Scientific, Bremen, Germany). The ionization method was ESI and operated in positive or negative ion mode. The capillary temperature was set at 275 °C. Samples were infused into the ESI source as MeOH solutions at a flow rate of 3 μL/min. The resolving power was 60,000 (fwhm) at *m*/*z* 400. Data acquisition and analysis were accomplished with Xcalibur software, version 3.0 (Thermo Fisher Scientific Inc.). Electron impact high-resolution MS measurements (EI-HRMS) were performed on a Thermo Q-Exactive GC Orbitrap mass spectrometer (Thermo Fisher Scientific, Bremen, Germany). The ion source temperature was set at 250 °C; the applied ionization energy was 70 eV. The resolving power was 60,000 (fwhm) at *m*/*z* 400. Data acquisition and analysis were accomplished with Xcalibur software version 4.0 (Thermo Fisher Scientific Inc.).

The logarithm of partition coefficient (*c* log *P*) was calculated using ChemAxon JChem-18.24 software. The original built-in method was slightly modified based on in-house measurements.

The log *D* values were determined by using a potentiometric titration method (T3-Sirius Instrument, Sirius Analytical Inc.). The pH-metric titrations were performed in the *n*-octanol–ionic strength adjusted water (0.15 M KCl aqueous solution) system.

#### 4-(Benzyloxy)-1-(2,5-dimethyl-1,2,3,4-tetrahydropyrimido­[1,6-*a*]­indol-7-yl)­pyridin-2­(1*H*)-one (**7**)

##### Step 1: 7-Bromo-2,5-dimethyl-1,2,3,4-tetrahydropyrimido­[1,6-*a*]­indole (**5**)

The solution of 1,3-dimethyl-4-piperidone (6.36 g, 49.7 mmol) and 4-bromophenylhydrazine hydrochloride (11.17 g, 50 mmol) in ethanol (100 mL) was refluxed for 1 h and evaporated to dryness. The solid residue was reduced to pieces with a spatula, cooled in an ice bath, and suspended in 70 mL of 28% hydrogen chloride in ethyl acetate while stirring. The so obtained suspension was stirred at reflux temperature for 70 min and then allowed to stand overnight at 8 °C in a refrigerator. The solid was filtered, washed with ethanol, then boiled in ethanol (10 mL), filtered, washed again with ethanol, and air-dried to obtain 14.15 g of solid. This crude hydrochloride salt was suspended in saturated NaHCO_3_ solution (80 mL), water (10 mL), and ethyl acetate (150 mL), the phases were separated, and the aqueous phase was extracted with ethyl acetate (2 × 50 mL). The combined organic phases were washed with brine (50 mL), dried over anhydrous Na_2_SO_4_, filtered, and concentrated in vacuo. The crude product (10.87 g) was crystallized from ethanol, and the crystals were filtered, washed with ethanol, and dried in vacuo at 45 °C to obtain 9.79 g (70%) of 7-bromo-2,5-dimethyl-1,2,3,4-tetrahydropyrimido­[1,6-*a*]­indole (**5**). HPLC-MS (ESI) Method 2 >99% 279.0 [M + H]^+^. ^1^H NMR (DMSO-*d*_6_, 500 MHz): δ 7.59 (d, 1H, *J* = 1.9 Hz), 7.29 (d, 1H, *J* = 8.6 Hz), 7.14 (dd, 1H, *J* = 1.9, 8.6 Hz), 4.68 (s, 2H), 2.90 (s, 4H), 2.45 (s, 3H), 2.12 (s, 3H). ^13^C NMR (DMSO-*d*_6_, 126 MHz): δ 132.9, 132.2, 129.9, 122.0, 119.7, 111.3, 110.6, 104.3, 65.4, 49.0, 40.4, 40.0, 39.8, 39.7, 39.5, 19.3, 7.6. EI-HRMS: calcd for C_13_H_15_N_2_Br [M]^+^, 278.04131; found, 278.04094; Δ = −1.3 ppm.

##### Step 2: 4-(Benzyloxy)-1-(2,5-dimethyl-1,2,3,4-tetrahydropyrimido­[1,6-*a*]­indol-7-yl)­pyridin-2­(1*H*)-one (**7**)

A mixture of 7-bromo-2,5-dimethyl-1,2,3,4-tetrahydropyrimido­[1,6-*a*]­indole (**5**, 2.79 g, 10 mmol), commercially available 4-(benzyloxy)­pyridin-2­(1*H*)-one (2 g, 10 mmol), copper­(I) iodide (1.9 g, 10 mmol), Cs_2_CO_3_ (4.24 g, 13 mmol), *trans*-*N*,*N*′-dimethylcyclohexane-1,2-diamine (1.58, 10 mmol), and toluene (110 mL) was stirred at room temperature for 1 h while nitrogen gas was bubbled through the mixture. Then the reaction flask was sealed with a rubber septum and immersed into an oil bath of 110 °C and the mixture was stirred overnight at this temperature. The reaction mixture was poured into a 90:10:1 mixture of dichloromethane, methanol and 28% NH_3_ in H_2_O (600 mL), washed with brine (3 × 200 mL), saturated NaHCO_3_ solution (100 mL) and brine again (2 × 100 mL), dried over anhydrous Na_2_SO_4_, filtered, and concentrated in vacuo. The brownish solid residue was crystallized first from isopropanole, then from ethanol to obtain 2.15 g of beige solid. The crude product was purified by flash column chromatography using Kieselgel 60 (0.040–0.063 mm) as adsorbent (Merck) and a gradient elution from 0 to 5% methanol in dichloromethane to afford 1.94 g (50%) of 4-(benzyloxy)-1-(2,5-dimethyl-1,2,3,4-tetrahydropyrimido­[1,6-*a*]­indol-7-yl)­pyridin-2­(1*H*)-one (**7**) as a free base. HPLC-MS (ESI) Method 2 >99% 400.2 [M + H]^+^. Preparation of the maleic acid salt. To a solution of 4-(benzyloxy)-1-(2,5-dimethyl-1,2,3,4-tetrahydropyrimido­[1,6-*a*]­indol-7-yl)­pyridin-2­(1*H*)-one (**7**, 1.94 g, 4.85 mmol) in a 10:1 mixture of dichloromethane and methanol, maleic acid (0.564 g, 4.85 mmol) was added, and the solution was concentrated. The solid residue was triturated with ethanol, filtered, washed with ethanol and diethyl ether, and dried in vacuo to yield **2**.18 g (42%) of 4-(benzyloxy)-1-(2,5-dimethyl-1,2,3,4-tetrahydropyrimido­[1,6-*a*]­indol-7-yl)­pyridin-2­(1*H*)-one (**7**) of maleic acid salt. HPLC-MS (ESI) Method 2 >99% 400.3 [M + H]^+^. HPLC purity (Method 1): 99.69%. ^1^H NMR (dichloromethane-*d*_2_, 500 MHz): δ 7.55 (d, 1H, *J* = 7.6 Hz, H-17), 7.45–7.49 (m, 2H, H-25, H-26), 7.41–7.45 (m, 2H, H-27, H-28), 7.39–7.41 (m, 2H, H-3, H-4), 7.35–7.39 (m, 1H, H-29), 7.01 (d, 1H, *J* = 8.3 Hz, H-6), 6.08 (dd, 1H, *J* = 2.7, 7.6 Hz, H-19), 5.96 (d, 1H, *J* = 2.7 Hz, H-20), 5.15 (s, 2H, H_2_-23), 4.97 (br s, 2H, H_2_-14), 3.19 (br s, 2H, H_2_-12), 3.04 (br t, 2H, *J* = 5.9 Hz, H_2_-11), 2.67 (s, 3H, H_3_-16), 2.16 (s, 3H, H_3_-10). ^13^C­{1H} NMR (dichloromethane-*d*_2_, 126 MHz): δ 166.8 [HOC=O maleic acid] 166.6 [C-21], 162.8 [C-18], 139.8 [C-17], 135.8 [C-24], 133.3 [C-2], 133.0 [C-5], 132.2 [−CH= maleic acid], 130.6 [C-8], 128.4 [C-27, C-28], 128.3 [C-1], 128.1 [C-29], 127.8 [C-25, C-26], 119.4 [C-6], 116.0 [C-3], 108.8 [C-4], 105.9 [C-7], 99.6 [C-19], 97.7 [C-20], 69.5 [C-23], 64.4 [C-14], 49.3 [C-12], 40.2 [C-16], 19.2 [C-11], 7.7 [C-10]. ESI-HRMS: calcd for C_25_H_26_O_2_N_3_ [M + H]^+^, 400.20195; found, 400.20192; Δ = −0.08 ppm.

The chemical stability measurement of compound **7** was performed. The samples were prepared from 1 mM DMSO stock solution diluted to 100 mM test concentration and incubated in clear glass vials with a total volume of 2 mL for 4 and 24 h at 37 °C. Analysis was performed by HPLC-MS.

#### 4-(Benzyloxy)-1-(2,3,4,5-tetrahydro-1*H*-[1,4]­diazepino­[1,2-*a*]­indol-9-yl)­pyridin-2­(1*H*)-one (**16**)

##### Step 1: *tert*-Butyl (3-Bromopropyl)­carbamate (**9**)

To a solution of 3-bromopropylamine hydrobromide (**8**, 10 g, 45.7 mmol) in water (24 mL) was added the solution of di-*tert*-butyl dicarbonate (5.1 g, 22.8 mmol) in dichloromethane (56 mL) followed by the dropwise addition of a solution of sodium hydroxide (3.64 g, 91 mmol) in water (23 mL) at room temperature with intensive stirring. The stirring was continued for two and half hours before the phases were separated, and the organic phase was washed with equal volumes (50 mL) of water, citric acid aqueous solution (10 w/v %), water, and brine in succession, dried over anhydrous Na_2_SO_4_, filtered, and concentrated in vacuo. The crude oily product (5.25 g, 96%) solidified on standing in the freezer overnight and was used without further purification. An analytical sample was prepared by flash column chromatography using Kieselgel 60 (0.040–0.063 mm) as adsorbent (Merck) and a 95:5 mixture of dichloromethane and acetone as the eluent. HPLC-MS (ESI) Method 3 >99% 222.9 [M-^*t*^Bu + MeCN + H]^+^. ^1^H NMR (DMSO-*d*_6_, 400 MHz): δ 6.91 (br s, 1H), 3.50 (t, 2H, *J* = 6.6 Hz), 2.99–3.07 (m, 2H), 1.90 (quin, 2H, *J* = 6.7 Hz), 1.38 (s, 9H). ^13^C NMR (DMSO-*d*_6_, 101 MHz): δ 156.1, 78.1, 38.9, 33.1, 32.8, 28.7. ESI-HRMS: calcd for C_8_H_16_O_2_NBrNa [M + Na]^+^, 260.02566; found, 260.02577; Δ = 0.4 ppm.

##### Step 2: Ethyl 5-Bromo-1-{3-[(*tert*-butoxycarbonyl)­amino]­propyl}-1*H*-indole-2-carboxylate (**11**)

To the solution of ethyl 5-bromoindole-2-carboxylate (10, 1 g, 3.73 mmol) in DMF (10 mL) sodium hydride (60%, 0.17 g, 4.25 mmol) was added under argon at 0 °C and stirred for 15 min at the same temperature. The solution of *tert*-butyl (3-bromopropyl)­carbamate (1.07 g, 4.5 mmol) in DMF (2 mL) as well as KI (10 mg, 0.06 mmol) was added to the reaction mixture at 0 °C and stirred overnight at RT. The reaction mixture was poured over ice (30 g) and water (30 mL), washed with 3 × 45 mL ethyl acetate, the combined organic phases were washed with 45 mL of brine, dried over anhydrous Na_2_SO_4_, filtered, and concentrated in vacuo. The crude product was dissolved in diethyl ether and precipitated by the addition of hexanes. Heating provided a solution and a tarry residue. The solution was decanted, its volume reduced to half of the original by evaporation, and crystallization was initiated by scratching the surface of the flask with a spatula. The solid was filtered, washed with hexanes, and air-dried to yield 1.17 g (74%) of ethyl 5-bromo-1-{3-[(*tert*-butoxycarbonyl)­amino]­propyl}-1*H*-indole-2-carboxylate (**11**). HPLC-MS (ESI) Method 3 99.86% 447.1 [M + Na]^+^. ^1^H NMR (DMSO-*d*_6_, 500 MHz): δ 7.92 (d, 1H, *J* = 1.9 Hz), 7.61 (d, 1H, *J* = 9.0 Hz), 7.44 (dd, 1H, *J* = 2.0, 8.9 Hz), 7.26 (s, 1H), 6.87 (br s, 1H), 4.55 (t, 2H, *J* = 7.2 Hz), 4.33 (q, 2H, *J* = 7.1 Hz), 2.91 (q, 2H, *J* = 6.6 Hz), 1.81 (br t, 2H, *J* = 7.1 Hz), 1.37 (s, 9H), 1.34 (t, 3H, *J* = 7.1 Hz). ^13^C NMR (DMSO-*d*_6_, 126 MHz): δ 160.7, 155.4, 137.2, 128.1, 127.3, 126.9, 124.4, 113.0, 112.9, 109.4, 77.5, 60.5, 42.2, 30.3, 28.1, 14.0. ESI-HRMS: calcd for C_19_H_26_O_4_N_2_Br [M + H]^+^, 425.10705; found, 425.10695; Δ = −0.2 ppm.

##### Step 3: 9-Bromo-2,3,4,5-tetrahydro-1*H*-[1,4]­diazepino­[1,2-*a*]­indol-1-one (**12**)

To the solution of ethyl 5-bromo-1-{3-[(*tert*-butoxycarbonyl)­amino]­propyl}-1*H*-indole-2-carboxylate (**11**, 1.17 g, 2.75 mmol) in DCM (12 mL) was added trifluoroacetic acid (3.6 mL, 47 mmol), and the solution was stirred for 90 min at room temperature. The volatiles were evaporated and chloroform (3 × 15 mL) was added to the residue and evaporated to obtain a solid. The latter was taken up in THF (22 mL) and MeOH (22 mL) and K_2_CO_3_ were added (2.4 g, 17.3 mmol), and the reaction mixture was stirred for 3 days at room temperature. Ethyl acetate (115 mL) and water (50 mL) were added to the reaction mixture, the phases were separated, and the organic phase was washed with brine (50 mL), then dried over anhydrous Na_2_SO_4_, filtered, and concentrated in vacuo. The residue was heated in ethanol (15 mL), and the solid was filtered at room temperature, washed with ethanol, and dried to yield 0.515 g (67%) of 9-bromo-2,3,4,5-tetrahydro-1*H*-[1,4]­diazepino­[1,2-*a*]­indol-1-one (**12**). HPLC-MS (ESI) Method 3 99.6% 279.0 [M + H]^+^. A further 84.8 mg crop (11%) of **12** could be obtained by the flash column chromatography of the evaporated ethanolic filtrate using Kieselgel 60 (0.040–0.063 mm) as the adsorbent (Merck) and a 4:1 mixture of dichloromethane and acetone as the eluent. ^1^H NMR (DMSO-*d*_6_, 800 MHz): δ 8.16 (br s, 1H), 7.84 (d, 1H, *J* = 1.7 Hz), 7.62 (d, 1H, *J* = 8.8 Hz), 7.37 (dd, 1H, *J* = 1.9, 8.8 Hz), 6.91 (s, 1H), 4.39 (t, 2H, *J* = 6.7 Hz), 3.11 (q, 2H, *J* = 6.1 Hz), 2.08 (quin, 2H, *J* = 6.5 Hz). ^13^C NMR (DMSO-*d*_6_, 201 MHz): δ 164.8, 136.2, 135.3, 127.7, 125.7, 123.6, 112.6, 112.2, 104.8, 41.1, 38.2, 28.9. ESI-HRMS: calcd for C_12_H_12_ON_2_Br [M + H]^+^, 279.01275; found, 279.01284; Δ = 0.3 ppm.

##### Step 4: 9-Bromo-2,3,4,5-tetrahydro-1*H*-[1,4]­diazepino­[1,2-*a*]­indole (**13**)

9-Bromo-2,3,4,5-tetrahydro-1*H*-[1,4]­diazepino­[1,2-*a*]­indol-1-one (**12**, 0.6 g, 2.15 mmol) was stirred in a toluene solution borane–dimethyl sulfide complex (2 M in toluene, 10.8 mL, 21.6 mmol) for 30 min at room temperature to obtain a solution, which was then stirred at 70 °C for 4 h. To the suspension ethanol (25 mL) and hydrochloric acid solution (6 N, 2.6 mL) was added and the solution was stirred for an hour at 80 °C and then allowed to cool to room temperature. To the mixture sodium hydroxide solution (7 M, 5.1 mL), water (20 mL) and ethyl acetate (50 mL) were added, the phases separated, and the aqueous phase was washed with ethyl acetate (30 mL). The combined organic phases were washed with water (20 mL) and brine (20 mL), then dried over anhydrous Na_2_SO_4_, filtered, and concentrated in vacuo. HPLC-MS analysis (Method 2) indicated that the residue contained a dihydro-derivative of the product (35%, presumably the indoline-derivative) alongside the desired product (49%) and the starting material (11%). To convert the dihydro side product into the target molecule, this mixture was dissolved in THF (5 mL) and a solution of DDQ (0.53 g, 2.3 mmol) in THF (5 mL) was added dropwise at 0 °C followed by stirring at room temperature for 72 h. To the reaction mixture sodium hydroxide solution (7 M, 15 mL) was added and the precipitate was dissolved by the addition of water and extracted with ethyl acetate (3 × 20 mL). The combined organic phases were washed with saturated sodium bicarbonate aqueous solution, water, and brine and then dried over anhydrous Na_2_SO_4_, filtered, and concentrated in vacuo to obtain 9-bromo-2,3,4,5-tetrahydro-1*H*-[1,4]­diazepino­[1,2-*a*]­indole (**13**) in HPLC-MS (ESI) Method 2. Purity was 85.9%, and 265.1 [M + H]^+^, which was used in the next step without further purification, while the yield was taken as quantitative. An analytical sample was prepared by flash column chromatography using Kieselgel 60 (0.040–0.063 mm) as the adsorbent (Merck) and first acetone, then a 95:5 mixture of DCM and methanolic solution of ammonia (7 M) as the eluent. HPLC-MS (ESI) Method 3 98.72% 265.0 [M + H]^+^. ^1^H NMR (DMSO-*d*_6_, 800 MHz): δ 7.63 (d, 1H, *J* = 1.8 Hz), 7.43 (d, 1H, *J* = 8.7 Hz), 7.17 (dd, 1H, *J* = 1.9, 8.7 Hz), 6.27 (s, 1H), 4.30 (br s, 2H), 3.93 (s, 2H), 3.05 (br s, 2H), 1.71 (br s, 2H). ^13^C NMR (DMSO-*d*_6_, 201 MHz): δ 135.5, 128.7, 122.7, 121.8, 111.3, 111.0, 98.5, 51.1, 46.0, 44.0, 31.1. ESI-HRMS: calcd for C_12_H_14_N_2_Br [M + H]^+^, 265.03349; found, 265.03339; Δ = −0.4 ppm.

##### Step 5: *tert*-Butyl 9-Bromo-4,5-dihydro-1*H*-[1,4]­diazepino­[1,2-*a*]­indole-2­(3*H*)-carboxylate (**14**)

To the solution of crude 9-bromo-2,3,4,5-tetrahydro-1*H*-[1,4]­diazepino­[1,2-*a*]­indole as prepared above (**13**) in dichloromethane (15 mL), di-*tert*-butyl dicarbonate (0.61 g, 2.8 mmol) and triethylamine (0.45 mL, 3.2 mmol) were added and the solution was stirred overnight at room temperature. The reaction mixture was diluted with dichloromethane (65 mL), washed with water (15 mL) and brine (20 mL), and then dried over anhydrous Na_2_SO_4_, filtered, and concentrated in vacuo. The crude product was purified by flash column chromatography using Kieselgel 60 (0.040–0.063 mm) as the adsorbent (Merck) and a 4:1 mixture of hexanes and ethyl acetate as the eluent to yield 0.368 g (46%, 2 steps) of *tert*-butyl 9-bromo-4,5-dihydro-1*H*-[1,4]­diazepino­[1,2-*a*]­indole-2­(3*H*)-carboxylate (**14**). HPLC-MS (ESI) Method 4 >99% 309.07 [M-^t^Bu + H]^+^. ^1^H NMR (DMSO-*d*_6_, 500 MHz): δ 7.65–7.72 (br s, 1H), 7.46 (d, 1H, *J* = 8.8 Hz), 7.21 (dd, 1H, *J* = 2.0, 8.7 Hz), 6.34 (s, 1H), 4.56 (br s, 2H), 4.38 (br s, 2H), 3.65 (br s, 2H), 1.74 (br s, 2H), 1.33 (s, 9H). ^13^C NMR (DMSO-*d*_6_, 126 MHz): δ 153.8, 140.2, 135.5, 128.5, 123.2, 122.2, 111.5, 111.2, 99.5/100.1, 78.9, 47.8/48.7, 43.6/44.2, 43.3/43.5, 28.6/29.2, 27.9. ESI-HRMS: calcd for C_17_H_22_O_2_N_2_Br [M + H]^+^, 365.08592; found, 365.08579; Δ = 0.4 ppm.

##### Step 6: *tert*-Butyl 9-[4-(Benzyloxy)-2-oxopyridin-1­(2*H*)-yl]-4,5-dihydro-1*H*-[1,4]­diazepino­[1,2-*a*]­indole-2­(3*H*)-carboxylate (**15**)

A mixture of 0.36 g (1 mmol) of *tert*-butyl 9-bromo-4,5-dihydro-1*H*-[1,4]­diazepino­[1,2-*a*]­indole-2­(3*H*)-carboxylate (**14**), 0.30 g (1.48 mmol) of commercially available 4-(benzyloxy)­pyridin-2­(1*H*)-one, 0.094 g (0.49 mmol) of copper­(I) iodide, 0.27 g (1.97 mmol) of K_2_CO_3_, 0.072 g (0.49 mmol) of 8-hydroxyquinoline, 1 g of molecular sieves (3 Å, 8–12 mesh), and 5 mL of dimethyl sulfoxide was stirred at room temperature while five evacuation and nitrogen refill cycles were applied. Then the reaction flask was sealed with a rubber septum, immersed into an oil bath of 130 °C, and the mixture was stirred for 46 h at this temperature. The reaction mixture was poured into a mixture of 13.5 mL of methanol and 1.5 mL of 28% NH_3_ in H_2_O and stirred for 15 min, then filtered, and the solid residue washed with methanol. 20 mL of brine was added to the filtrate, the mixture was filtered, and the solid residue was thoroughly washed with ethyl acetate. The liquid phases were separated, the aqueous layer was washed with ethyl acetate, and then the combined organic phases were washed with brine, dried over anhydrous Na_2_SO_4_, filtered, and concentrated in vacuo. The crude product was purified by flash column chromatography using Kieselgel 60 (0.040–0.063 mm) as the adsorbent (Merck) and a 95:5 mixture of dichloromethane and methanol as the eluent to yield 0.42 g (89%) of *tert*-butyl 9-[4-(benzyloxy)-2-oxopyridin-1­(2*H*)-yl]-4,5-dihydro-1*H*-[1,4]­diazepino­[1,2-*a*]­indole-2­(3*H*)-carboxylate (**15**) as a light greenish solid. HPLC-MS (ESI) Method 3 97.91% 486.3 [M + H]^+^. ^1^H NMR (DMSO-*d*_6_, 500 MHz): δ 7.51–7.58 (m, 2H), 7.45–7.49 (m, 2H), 7.40–7.45 (m, 3H), 7.34–7.39 (m, 1H), 7.02 (dd, 1H, *J* = 2.1, 8.7 Hz), 6.40 (s, 1H), 6.06 (dd, 1H, *J* = 2.7, 7.6 Hz), 5.96 (d, 1H, *J* = 2.7 Hz), 5.14 (s, 2H), 4.60 (br s, 2H), 4.43 (br s, 2H), 3.67 (br s, 2H), 1.77 (br s, 2H), 1.34 (s, 9H). ^13^C NMR (DMSO-*d*_6_, 126 MHz): δ 166.6, 162.8, 153.7, 140.0, 139.8, 135.9, 135.8, 132.6, 128.4, 128.1, 127.8, 126.5, 120.0, 118.1/118.2, 109.5, 100.2/100.8, 99.6, 97.7, 78.8, 69.5, 47.8/48.6, 43.7/44.2, 43.4/43.5, 28.8/29.3, 27.9. ESI-HRMS: calcd for C_29_H_32_O_4_N_3_ [M + H]^+^, 486.23873; found, 486.23853; Δ = −0.4 ppm.

##### Step 7: 4-(Benzyloxy)-1-(2,3,4,5-tetrahydro-1*H*-[1,4]­diazepino­[1,2-*a*]­indol-9-yl)­pyridin-2­(1*H*)-one (**16**)

A mixture of 0.42 g (0.86 mmol) of *tert*-butyl 9-[4-(benzyloxy)-2-oxopyridin-1­(2*H*)-yl]-4,5-dihydro-1*H*-[1,4]­diazepino­[1,2-*a*]­indole-2­(3*H*)-carboxylate (**15**), methanol (10 mL), and a 20% hydrogen chloride solution in ethyl acetate (5 mL) was stirred at room temperature for 2 h and then evaporated. The residue was taken up in saturated NaHCO_3_ solution (20 mL) and a 9:1 mixture of ethyl acetate and isopropanol (30 mL), the separated solid was filtered, the phases separated, and the aqueous phase extracted with the same type of solvent mixture three times. The solid and the organic phases were combined and evaporated. The residue was triturated with diethyl ether, ultrasonically agitated, and filtered, then triturated again with 10 mL of hydrochloric acid solution (1 N), filtered, and the solid washed consecutively with a sodium hydroxide solution (1 N), methanol, ethanol, ethyl acetate, and diethyl ether. The crude product was purified by flash column chromatography using Kieselgel 60 (0.040–0.063 mm) as the adsorbent (Merck) and a 95:5:1 mixture of dichloromethane, methanol, and 28% NH_3_ in H_2_O solution as the eluent to obtain 0.12 g of a yellowish white solid. The so-obtained product was converted to its hydrochloride salt by dissolving it in methanol and a hydrogen chloride solution in methanol (1.25 M); then, the solution was evaporated. The residue was triturated in diethyl ether, filtered, and dried to yield 0.116 g (35%) of 4-(benzyloxy)-1-(2,3,4,5-tetrahydro-1*H*-[1,4]­diazepino­[1,2-*a*]­indol-9-yl)­pyridin-2­(1*H*)-one monohydrochloride (**16**) as a beige solid. LCMS-MS (ESI) Method 2 >99% 386.2 [M + H]^+^. HPLC purity (Method 1): 98.82%. ^1^H NMR (dichloromethane-*d*_2_, 500 MHz): δ 9.44 (br s, 2H, NH^2+^-11), 7.61 (d, 1H, *J* = 8.9 Hz, H-4), 7.56 (d, 1H, *J* = 7.6 Hz, H-16), 7.51 (d, 1H, *J* = 2.0 Hz, H-3), 7.45–7.49 (m, 2H, H-24, H-25), 7.40–7.45 (m, 2H, H-26, H-27), 7.35–7.40 (m, 1H, H-28), 7.12 (dd, 1H, *J* = 2.1, 8.8 Hz, H-2), 6.70 (s, 1H, H-7), 6.08 (dd, 1H, *J* = 2.7, 7.6 Hz, H-18), 5.97 (d, 1H, *J* = 2.7 Hz, H-19), 5.14 (s, 2H, H_2_-22), 4.56 (br s, 2H, H_2_-10), 4.49 (br s, 2H, H_2_-12), 3.38–3.43 (m, 2H, H_2_-14), 2.01 (br s, 2H, H_2_-13). ^13^C NMR (dichloromethane-*d*_2_, 126 MHz): δ 166.7 [C-20], 162.8 [C-17], 139.7 [C-16], 136.0 [C-6], 135.8 [C-23], 133.7 [C-8], 133.0 [C-1], 128.4 [C-26, C-27], 128.1 [C-28], 127.8 [C-24, C-25], 126.1 [C-5], 121.1 [C-2], 118.6 [C-3], 109.8 [C-4], 104.2 [C-7], 99.7 [C-18], 97.7 [C-19], 69.5 [C-22], 48.0 [C-14], 42.8 [C-10], 42.6 [C-12], 26.4 [C-13]. ESI-HRMS: calcd for C_24_H_24_O_2_N_3_ [M + H]^+^, 386.18630; found, 386.18628; Δ = −0.06 ppm.

The preparation of 4,5,11,11a-tetrahydro-1*H*-[1,4]­diazepino­[1,7-*a*]­indol-2­(3*H*)-one (**24**) from 2-nitrophenylacetic acid (**17**) in seven steps from our laboratory is described in the literature.^18^

#### 4-(Benzyloxy)-1-(2,3,4,5-tetrahydro-1*H*-[1,4]­diazepino­[1,7-*a*]­indol-9-yl)­pyridin-2­(1*H*)-one (**30**) Maleic Acid Salt

##### Step 1: 2,3,4,5,11,11a-Hexahydro-1*H*-[1,4]­diazepino­[1,7-*a*]­indole (**25**)

To the suspension of 4,5,11,11a-tetrahydro-1*H*-[1,4]­diazepino­[1,7-*a*]­indol-2­(3*H*)-one (**24**, 4.74 g, 23.44 mmol) in tetrahydrofuran (230 mL), borane dimethyl sulfide complex solution (2 M in THF, 35 mL, 70 mmol) was added dropwise. The solution was heated for 22 h at reflux temperature before cooling down. Ethanol (115 mL) was carefully added and after the gas evolution subsided and the mixture was evaporated before redissolving in ethanol (160 mL) and hydrochloric acid solution (1 N, 100 mL). The solution was heated for an hour at reflux temperature, cooled down, and the organic solvent removed by rotary evaporation. The pH of the aqueous residue was adjusted to 13 by the addition of sodium hydroxide solution (1 N, 170 mL) and the mixture was washed with dichloromethane (3 × 160 mL). The combined organic phases were washed with brine (160 mL), dried over anhydrous Na_2_SO_4_, filtered, and concentrated in vacuo. The crude product was purified by column chromatography using Kieselgel 60 (0.040–0.063 mm) as the adsorbent (Merck) and a 95:5:1 mixture of dichloromethane, methanol, and 28% NH_3_ in H_2_O as the eluent to yield 3.42 g (77%) of 2,3,4,5,11,11a-hexahydro-1*H*-[1,4]­diazepino­[1,7-*a*]­indole (**25**) as a yellow oil. HPLC-MS (ESI) Method 3 97.39% 189.1 [M + H]^+^. ^1^H NMR (DMSO-*d*_6_, 400 MHz): δ 6.89–7.00 (m, 2H), 6.49 (t, 1H, *J* = 7.1 Hz), 6.29 (d, 1H, *J* = 7.6 Hz), 3.87 (dq, 1H, *J* = 2.4, 9.2 Hz), 3.34–3.42 (m, 1H), 3.15 (dd, 1H, *J* = 9.3, 15.9 Hz), 2.94–3.03 (m, 1H), 2.78–2.93 (m, 3H), 2.62–2.73 (m, 1H), 2.56 (dd, 1H, *J* = 8.7, 15.9 Hz), 1.79–1.89 (m, 1H), 1.61–1.76 (m, 1H). ^13^C NMR (DMSO-*d*_6_, 101 MHz): δ 152.5, 128.1, 127.1, 123.4, 116.1, 105.5, 63.2, 50.4, 47.5, 47.1, 38.8, 36.3. EI-HRMS: calcd for C_12_H_16_N_2_ [M]^+^, 188.13080; found, 188.13092; Δ = 0.64 ppm.

##### Step 2: *tert*-Butyl 1,2,4,5,11,11a-Hexahydro-3*H*-[1,4]­diazepino­[1,7-*a*]­indole-3-carboxylate (**26**)

To a solution of 2,3,4,5,11,11a-hexahydro-1*H*-[1,4]­diazepino­[1,7-*a*]­indole (**25**, 3.4 g, 18 mmol) in dichloromethane (170 mL), triethylamine (3.8 mL, 27.09 mmol) was added, then a solution of di-*tert*-butyl-dicarbonate (4.73 g, 21.67 mmol) in dichloromethane (25 mL) was added dropwise at 0 °C. The reaction mixture was stirred at room temperature for 3.5 h, then saturated NaHCO_3_ solution (150 mL) was added, the phases were separated, and the water phase was extracted with dichloromethane (2 × 50 mL). The combined organic phases were washed with brine (50 mL), dried over anhydrous Na_2_SO_4_, filtered, and concentrated in vacuo. The crude product was purified by flash column chromatography using Kieselgel 60 (0.040–0.063 mm) as the adsorbent (Merck) and a 4:1 mixture of hexane and ethyl acetate as the eluent to yield 4.88 g (94%) of *tert*-butyl 1,2,4,5,11,11a-hexahydro-3*H*-[1,4]­diazepino­[1,7-*a*]­indole-3-carboxylate (**26**). HPLC-MS (ESI) Method 2 >99% 289.2 [M + H]^+^. ^1^H NMR (DMSO-*d*_6_, 500 MHz): δ 6.94–7.00 (m, 2H), 6.55 (t, 1H, *J* = 7.3 Hz), 6.45 (d, 1H, *J* = 8.0 Hz), 3.53–3.69 (m, 4H), 3.44–3.53 (m, 1H), 3.23–3.31 (m, 1H), 3.16 (dd, 1H, *J* = 8.9, 15.7 Hz), 2.72–2.83 (m, 1H), 2.56 (dd, 1H, *J* = 9.6, 15.6 Hz), 1.91–2.03 (m, 1H), 1.67–1.80 (m, 1H), 1.39 (s, 9H). ^13^C NMR (DMSO-*d*_6_, 126 MHz): δ 154.3, 152.4, 128.2, 127.1, 123.6, 117.1, 106.5, 78.5, 64.0/64.6, 47.3, 45.2/45.7, 45.2, 44.3/44.9, 35.9/36.0, 35.2/35.5, 28.0. ESI-HRMS: calcd for C_17_H_25_O_2_N_2_ [M + H]^+^, 289.19105; found, 289.19019; Δ = −2.99 ppm.

##### Step 3: *tert*-Butyl 9-Bromo-1,2,4,5,11,11a-hexahydro-3*H*-[1,4]­diazepino­[1,7-*a*]­indole-3-carboxylate (**27**)

To a solution of *tert*-butyl 1,2,4,5,11,11a-hexahydro-3*H*-[1,4]­diazepino­[1,7-*a*]­indole-3-carboxylate (**26**, 4.87 g, 16.9 mmol) in acetonitrile (160 mL) was added dropwise a solution of *N*-bromo-succinimide (3 g, 16.9 mmol) in acetonitrile (45 mL) at 0 °C. The reaction mixture was stirred at room temperature for 2.5 h, then 1 mL of acetone was added, stirring was continued for 5 min, and then the reaction mixture was concentrated in vacuo. The residue was purified by column chromatography using Kieselgel 60 (0.040–0.063 mm) as the adsorbent (Merck) and a 95:5 mixture of cyclohexane and acetone as the eluent to yield 4.96 g (80%) of *tert*-butyl 9-bromo-1,2,4,5,11,11a-hexahydro-3*H*-[1,4]­diazepino­[1,7-*a*]­indole-3-carboxylate (**27**). HPLC-MS (ESI) Method 2 >99% 367.1 [M + H]^+^. ^1^H NMR (DMSO-*d*_6_, 500 MHz): δ 7.07–7.13 (m, 2H), 6.39 (d, 1H, *J* = 8.9 Hz), 3.53–3.74 (m, 1H), 3.40–3.51 (m, 1H), 3.21–3.27 (m, 1H), 3.18 (dd, 2H, *J* = 9.0, 16.2 Hz), 2.76–2.86 (m, 1H), 2.58 (dd, 1H, *J* = 9.1, 16.2 Hz), 1.87–1.98 (m, 1H), 1.65–1.78 (m, 1H), 1.38 (s, 9H). ^13^C NMR (DMSO-*d*_6_, 126 MHz): δ 154.3/154.2, 151.50/151.47, 131.1, 129.5, 126.4, 108.0/107.9, 107.62/107.60, 78.5, 64.5/63.9, 46.8, 45.4/44.9, 44.9/44.3, 35.6/35.2, 35.52/35.51, 28.0. ESI-HRMS: calcd for C_17_H_24_O_2_N_2_Br [M + H]^+^, 367.10157; found, 367.10068; Δ = −2.42 ppm.

##### Step 4: *tert*-Butyl 9-Bromo-1,2,4,5-tetrahydro-3*H*-[1,4]­diazepino­[1,7-*a*]­indole-3-carboxylate (**28**)

To a solution of 9-bromo-1,2,4,5,11,11a-hexahydro-3*H*-[1,4]­diazepino­[1,7-*a*]­indole-3-carboxylate (**27**, 4.95 g, 13.5 mmol) in tetrahydrofuran (120 mL) was added 5,6-dicyano-2,3-dichloro-1,4-benzoquinone (3.37 g, 14.8 mmol) in small portions at 0 °C; then the reaction mixture was stirred at 0 °C for 45 min. Aqueous sodium hydroxide (2 N, 320 mL) was added to the reaction mixture, the phases were separated, and the aqueous phase was extracted with ethyl acetate (3 × 120 mL). The combined organic phases were washed with water (2 × 60 mL) and brine (120 mL), then dried over anhydrous Na_2_SO_4_, filtered, and concentrated in vacuo. The crude product was dissolved in ethanol (25 mL) at reflux temperature, and then the solid product that separated at room temperature was filtered, washed with ethanol and hexane, and dried to yield 4.05 g (82%) of *tert*-butyl 9-bromo-1,2,4,5-tetrahydro-3*H*-[1,4]­diazepino­[1,7-*a*]­indole-3-carboxylate (**28**). HPLC-MS (ESI) Method 2 >99% 387.0 [M + Na]^+^. ^1^H NMR (DMSO-*d*_6_, 500 MHz): δ 7.62 (d, 1H, *J* = 1.9 Hz), 7.42 (d, 1H, *J* = 8.8 Hz), 7.18 (dd, 1H, *J* = 2.0, 8.7 Hz), 6.27 (s, 1H), 4.30 (br s, 2H), 3.59–3.67 (m, 2H), 3.51–3.58 (m, 2H), 2.97–3.07 (m, 2H), 1.44 (s, 9H). ^13^C NMR (DMSO-*d*_6_, 126 MHz): δ 142.6, 135.7, 128.9, 122.6, 121.5, 111.32, 111.26, 99.8, 79.2, 47.5/46.7, 46.2/45.4, 45.8/45.7, 29.5/29.2, 28.0. ESI-HRMS: calcd for C_17_H_22_O_2_N_2_Br [M + H]^+^, 365.08592; found, 365.08580; Δ = −0.32 ppm.

##### Step 5: *tert*-Butyl 9-[4-(Benzyloxy)-2-oxopyridin-1­(2*H*)-yl]-1,2,4,5-tetrahydro-3*H*-[1,4]­diazepino­[1,7-*a*]­indole-3-carboxylate (**29**)

A mixture of *tert*-butyl 9-bromo-1,2,4,5-tetrahydro-3*H*-[1,4]­diazepino­[1,7-*a*]­indole-3-carboxylate (**28**, 0.84 g, 1.6 mmol), 4-benzyloxy-2­(1*H*)-pyridone (0.33 g, 1.6 mmol), copper­(I) iodide (0.31 g, 1.6 mmol), Cs_2_CO_3_ (0.74 g, 2.25 mmol), *trans*-*N*,*N*′-dimethylcyclohexan-1,2-diamine (0.25 mL, 1.6 mmol), and toluene (22 mL) was stirred at room temperature for 1 h while nitrogen gas was bubbled through the mixture. Then the reaction flask was sealed with a rubber septum and immersed into an oil bath of 110 °C, and the mixture was stirred overnight at this temperature. The reaction mixture was concentrated in vacuo, saturated aqueous ammonium chloride solution (22 mL) was added to the residue, and the suspension was stirred at room temperature for 4 h. The solid product was filtered, washed with saturated aqueous ammonium chloride solution (10 mL) and water (10 mL), and then air-dried. The crude product was purified by flash column chromatography using Kieselgel 60 (0.040–0.063 mm) as the adsorbent (Merck) and first dichloromethane and then a 98:2 mixture of dichloromethane and methanol as the eluent. The so-obtained solid was triturated with diethyl ether, stirred overnight, filtered, washed with diethyl ether, and dried to yield 0.65 g (84%) of *tert*-butyl 9-[4-(benzyloxy)-2-oxopyridin-1­(2*H*)-yl]-1,2,4,5-tetrahydro-3*H*-[1,4]­diazepino­[1,7-*a*]­indole-3-carboxylate (**29**). HPLC-MS (ESI) Method 2 >99% 486.2 [M + H]^+^. ^1^H NMR (DMSO-*d*_6_, 800 MHz): δ 7.4–7.5 (m, 2H), 7.4–7.4 (m, 5H), 7.36 (br d, 1H, *J* = 7.3 Hz), 7.05 (d, 1H, *J* = 8.8 Hz), 6.32 (s, 1H), 6.06 (dd, 1H, *J* = 2.7, 7.5 Hz), 5.97 (d, 1H, *J* = 2.7 Hz), 5.10 (s, 2H), 4.3–4.3 (m, 2H), 3.7–3.7 (m, 2H), 3.6–3.7 (m, 2H), 3.07 (dd, 2H, *J* = 4.3, 5.5 Hz), 1.50 (s, 9H). ^13^C NMR (DMSO-*d*_6_, 126 MHz): δ 167.0, 163.6, 142.3, 139.2, 136.3, 135.5, 132.8, 128.48/128.46, 128.15/128.13, 127.59/127.57, 127.4, 119.5, 117.8, 109.1, 101.0, 100.2, 98.1, 79.7, 69.8, 48.1/47.3, 46.9/46.2, 46.4/46.3, 30.3/30.0, 28.23/28.22. ESI-HRMS: calcd for C_29_H_32_O_4_N_3_ [M + H]^+^, 486.23873; found, 486.23761; Δ = −2.31 ppm.

##### Step 6: 4-(Benzyloxy)-1-(2,3,4,5-tetrahydro-1*H*-[1,4]­diazepino­[1,7-*a*]­indol-9-yl)­pyridin-2­(1*H*)-one (**30**) Maleic Acid Salt

A mixture of *tert*-butyl 9-[4-(benzyloxy)-2-oxopyridin-1­(2*H*)-yl]-1,2,4,5-tetrahydro-3*H*-[1,4]­diazepino­[1,7-*a*]­indole-3-carboxylate (**29**, 0.65 g, 1.34 mmol), ethyl acetate (20 mL), and hydrogen chloride solution in ethyl acetate (20%, 6 mL) was stirred at room temperature for 2 h; then a further amount of hydrogen chloride solution in ethyl acetate (20%, 6 mL) was added and the mixture was stirred at room temperature overnight. The solid product was filtered, washed with ethyl acetate and diethyl ether, and dried to yield 0.62 g (>100%) of 4-(benzyloxy)-1-(2,3,4,5-tetrahydro-1*H*-[1,4]­diazepino­[1,7-*a*]­indol-9-yl)­pyridin-2­(1*H*)-one (**30**) hydrochloride salt. HPLC-MS (ESI) Method 5 99.15% 386.2 [M + H]^+^. To 0.58 g (1.37 mmol) of this hydrochloride salt, 5% aqueous NaHCO_3_ solution (14 mL) and a 9:1 mixture of dichloromethane and 2-propanol (40 mL) were added, and the mixture stirred at room temperature for 10 min; then the phases were separated. The aqueous phase was washed with portions of a 9:1 mixture of dichloromethane and 2-propanol (2 × 12 mL). The combined organic phases were washed with brine (15 mL), then dried over anhydrous Na_2_SO_4_, filtered, concentrated in vacuo, and dried to yield 0.44 g (83%) of 4-(benzyloxy)-1-(2,3,4,5-tetrahydro-1*H*-[1,4]­diazepino­[1,7-*a*]­indol-9-yl)­pyridin-2­(1*H*)-one (**30**) as a free base. HPLC-MS (ESI) Method 2 98.99% 386.2 [M + H]^+^. Preparation of the maleic acid salt: to a solution of the free base (0.21 g, 0.54 mmol) in a 10:1 mixture of dichloromethane and methanol, maleic acid (0.078 g, 0.67 mmol) was added, and the reaction mixture was concentrated. The solid residue was triturated with ethanol, filtered, washed with ethanol and diethyl ether, and then dried to yield 0.218 g (80%) of 4-(benzyloxy)-1-(2,3,4,5-tetrahydro-1*H*-[1,4]­diazepino­[1,7-*a*]­indol-9-yl)­pyridin-2­(1*H*)-one (**30**) maleic acid salt. HPLC-MS (ESI) Method 2 >99% 386.2 [M + H]^+^. HPLC purity (Method 1) 99.66%. ^1^H NMR (500 MHz, DMSO-*d*_6_): δ 8.80–9.15 (br m, 2H, NH^2+^-11), 7.51–7.56 (m, 2H, H-5, H-16), 7.45–7.49 (m, 2H, H-24, H-25), 7.41–7.45 (m, 2H, H-26, H-27), 7.35–7.40 (m, 1H, H-28), 7.05 (dd, *J* = 8.7, 2.1 Hz, 1H, H-18), 6.43 (s, 1H, H-7), 6.08 (dd, *J* = 7.6, 2.7 Hz, 1H, H-18), 6.04 (s, 2H, –CH= maleic acid), 5.95 (d, *J* = 2.7 Hz, 1H, H-19), 5.14 (s, 2H, H_2_-22), 4.50–4.62 (br m, 2H, H_2_-13), 3.20–3.45 (m, 6H, H_2_-9, H_2_-12, H_2_-14). ^13^C NMR (DMSO-*d*_6_, 126 MHz): δ 167.0 [HOC=O maleic acid], 166.6 [C-20], 162.8 [C-17], 140.4 [C-8], 139.7 [C-16], 135.8 [C-23], 135.7 [C-3], 135.5 [−CH= maleic acid], 133.0 [C-4], 128.4 [C-26, C-27], 128.1 [C-28], 127.8 [C-24, C-25], 126.9 [C-1], 119.9 [C-6], 118.0 [C-2], 109.4 [C-5], 100.7 [C-7], 99.6 [C-18], 97.7 [C-19], 69.5 [C-22], 46.8 [C-12], 45.5 [C-14], 40.8 [C-13], 24.5 [C-9]. ESI-HRMS: calcd for C_24_H_24_O_2_N_3_ [M + H]^+^, 386.18630; found, 386.18636; Δ = 0.15 ppm.

#### 4-[(5-Chloropyridin-2-yl)­methoxy]-1-[3-(propan-2-yl)-2,3,4,5-tetrahydro-1*H*-[1,4]­diazepino­[1,7-*a*]­indol-9-yl]­pyridin-2­(1*H*)-one (**37**) Maleic Acid Salt

The preparation of intermediate 4-[(5-chloropyridin-2-yl)­methoxy]­pyridin-2­(1*H*)-one (**34**) from 4-nitropyridine *N*-oxide (**31**) in two steps from our laboratory is described in the literature.^15^

##### Step 1: *tert*-Butyl 9-{4-[(5-Chloropyridin-2-yl)­methoxy]-2-oxopyridin-1­(2*H*)-yl}-1,2,4,5-tetrahydro-3*H*-[1,4]­diazepino­[1,7-*a*]­indole-3-carboxylate (**35**)

A mixture of *tert*-butyl 9-bromo-1,2,4,5-tetrahydro-3*H*-[1,4]­diazepino­[1,7-*a*]­indole-3-carboxylate (**28**, 0.92 g, 2.52 mmol), 4-[(5-chloropyridin-2-yl)­methoxy]­pyridin-2­(1*H*)-one (**34**, 0.64 g, 2.7 mmol), copper­(I) iodide (0.51 g, 2.7 mmol), Cs_2_CO_3_ (1.14 g, 3.5 mmol), *trans*-*N*,*N*′-dimethylcyclohexan-1,2-diamine (0.43 mL, 2.7 mmol), and toluene (50 mL) was stirred at room temperature for 1 h while nitrogen gas was bubbled through the mixture. Then the reaction flask was sealed with a rubber septum, immersed into an oil bath of 110 °C, and the mixture was stirred overnight at this temperature. The reaction mixture was poured into a 9:1:0.1 mixture of dichloromethane, methanol, and 28% NH_3_ in H_2_O (165 mL), and the phases were separated. The organic phase was washed with portions of brine (each 30 mL) until the separated aqueous phase remained colorless, then dried over anhydrous Na_2_SO_4_, filtered, and concentrated in vacuo. The crude product was purified by flash column chromatography using Kieselgel 60 (0.040–0.063 mm) as the adsorbent (Merck) and first dichloromethane, then a 98:2 mixture of dichloromethane and methanol as the eluent to yield 0.86 g (66%) of *tert*-butyl 9-{4-[(5-chloropyridin-2-yl)­methoxy]-2-oxopyridin-1­(2*H*)-yl}-1,2,4,5-tetrahydro-3*H*-[1,4]­diazepino­[1,7-*a*]­indole-3-carboxylate (**35**) as an off-white solid. HPLC-MS (ESI) Method 2 >99% 521.2 [M + H]^+^. ^1^H NMR (DMSO-*d*_6_, 800 MHz): δ 8.67 (d, 1H, *J* = 2.4 Hz), 8.02 (dd, 1H, *J* = 2.5, 8.3 Hz), 7.61 (d, 1H, *J* = 8.4 Hz), 7.56 (d, 1H, *J* = 7.6 Hz), 7.50 (d, 1H, *J* = 8.7 Hz), 7.37 (d, 1H, *J* = 1.9 Hz), 6.99 (dd, 1H, *J* = 1.9, 8.7 Hz), 6.33 (s, 1H), 6.10 (dd, 1H, *J* = 2.8, 7.6 Hz), 5.94 (d, 1H, *J* = 2.7 Hz), 5.22 (s, 2H), 4.35 (br s, 2H), 3.64 (br s, 2H), 3.56 (br s, 2H), 3.05 (br s, 2H), 1.46 (s, 9H). ^13^C NMR (DMSO-*d*_6_, 201 MHz): δ 166.3, 162.7, 154.1, 147.8/147.7, 142.4, 139.9, 136.8, 136.0, 132.6, 130.4, 126.9, 123.4, 119.4, 117.5, 109.3, 100.5, 99.4, 97.9, 79.2, 69.6, 46.9/47.8, 45.7/46.4, 45.7/45.9, 29.3/29.6. ESI-HRMS: calcd for C_28_H_30_O_4_N_4_Cl [M + H]^+^, 521.19501; found, 521.19501; Δ = 0 ppm.

##### Step 2: 4-[(5-Chloropyridin-2-yl)­methoxy]-1-(2,3,4,5-tetrahydro-1*H*-[1,4]­diazepino­[1,7-*a*]­indol-9-yl)­pyridin-2­(1*H*)-one (**36**)

*Synthesis of the Dihydrochloride Salt*. A mixture of *tert*-butyl 9-{4-[(5-chloropyridin-2-yl)­methoxy]-2-oxopyridin-1­(2*H*)-yl}-1,2,4,5-tetrahydro-3*H*-[1,4]­diazepino­[1,7-*a*]­indole-3-carboxylate (**35**, 1.32 g, 2.54 mmol), methanol (15 mL), hydrogen chloride solution in methanol (1.25 M, 15 mL), and hydrogen chloride solution in ethyl acetate (20%, 30 mL) was stirred at room temperature overnight. The solid product was filtered, washed with diethyl ether, and dried to yield 1.26 g (100%) of 4-[(5-chloropyridin-2-yl)­methoxy]-1-(2,3,4,5-tetrahydro-1*H*-[1,4]­diazepino­[1,7-*a*]­indol-9-yl)­pyridin-2­(1*H*)-one (**36**) dihydrochloride salt. HPLC-MS (ESI) Method 2 >99% 421.1 [M + H]^+^. HPLC purity (Method 1) 99.50%. ^1^H NMR (500 MHz, DMSO-*d*_6_): δ 9.69 (br s, 2H, NH^2+^-11), 8.68 (d, *J* = 2.5 Hz, 1H, H-27), 8.03 (dd, *J* = 8.2, 2.5 Hz, 1H, H-26), 7.61 (d, *J* = 8.4 Hz, 1H, H-24), 7.58 (d, *J* = 7.6 Hz, 1H, H-16), 7.54 d (d, *J* = 8.8 Hz, 1H, H-5), 7.42 (d, *J* = 2.0 Hz, 1H, H-2), 7.05 (dd, *J* = 8.8, 2.2 Hz, 1H, H-6), 6.42 (s, 1H, H-7), 6.15 (dd, *J* = 7.5, 2.6 Hz, 1H, H-18), 5.98 (d, *J* = 2.7 Hz, 1H, H-19), 5.23 (s, 2H, H_2_-22), 4.62–4.70 (m, 2H, H_2_-13), 3.31–3.40 (m, 4H, H_2_-9, H_2_-14), 3.23–3.30 (br m, 2H, H_2_-12). ^13^C NMR (DMSO-*d*_6_, 126 MHz): δ 166.4 [C-20], 162.7 [C-17], 154.0 [C-23], 147.7 [C-27], 140.6 [C-8], 139.9 [C-16], 136.9 [C-26], 135.7 [C-3], 132.9 [C-4], 130.4 [C-28], 126.9 [C-1], 123.4 [C-24], 119.8 [C-6], 117.9 [C-2], 109.5 [C-5], 100.7 [C-7], 99.5 [C-18], 97.9 [C-19], 69.7 [C-22], 46.6 [C-14], 45.3 [C-12], 40.6 [C-13], 24.3 [C-9]. ESI-HRMS: calcd for C_23_H_22_O_2_N_4_Cl [M + H]^+^, 421.14258; found, 421.14044; Δ = −5.08 ppm.

*Synthesis of the Free Base*. A mixture of *tert*-butyl 9-{4-[(5-chloropyridin-2-yl)­methoxy]-2-oxopyridin-1­(2*H*)-yl}-1,2,4,5-tetrahydro-3*H*-[1,4]­diazepino­[1,7-*a*]­indole-3-carboxylate (**35**, 4.44 g, 8.52 mmol), ethyl acetate (130 mL), and hydrogen chloride solution in ethyl acetate (20%, 20 mL) was stirred at room temperature for 2 h. The solid product was filtered, washed with ethanol and diethyl ether, and dried. To the crude product NaHCO_3_ solution (5%, 85 mL) and a 9:1 mixture of dichloromethane and 2-propanol (250 mL) were added, and the phases were separated. The aqueous phase was extracted with a 9:1 mixture of dichloromethane and 2-propanol (2 × 80), and the combined organic phases were washed with brine (80 mL), then dried over anhydrous Na_2_SO_4_, filtered, and concentrated in vacuo. Diethyl ether (50 mL) was added to the residue and the mixture was stirred at room temperature overnight. The solid product was filtered, washed with diethyl ether, and dried to yield 3.39 g (95%) of 4-[(5-chloropyridin-2-yl)­methoxy]-1-(2,3,4,5-tetrahydro-1*H*-[1,4]­diazepino­[1,7-*a*]­indol-9-yl)­pyridin-2­(1*H*)-one (**36**) as a free base. HPLC-MS (ESI) Method 2 >99% 421.2 [M + H]^+^.

##### Step 3: 4-[(5-Chloropyridin-2-yl)­methoxy]-1-[3-(propan-2-yl)-2,3,4,5-tetrahydro-1*H*-[1,4]­diazepino­[1,7-*a*]­indol-9-yl]­pyridin-2­(1*H*)-one (**37**) Maleic Acid Salt

A mixture of 4-[(5-chloropyridin-2-yl)­methoxy]-1-(2,3,4,5-tetrahydro-1*H*-[1,4]­diazepino­[1,7-*a*]­indol-9-yl)­pyridin-2­(1*H*)-one free base as described above (**36**, 0.57 g, 1.36 mmol), acetonitrile (60 mL), K_2_CO_3_ (0.38 g, 2.72 mmol), and 2-iodopropane (1.36 mL, 13.6 mmol) was stirred at reflux temperature for 45 h. The reaction mixture was concentrated, the residue was triturated with water (20 mL), and the solid product was filtered and washed with water. The crude product was purified by column chromatography using Kieselgel 60 (0.040–0.063 mm) as the adsorbent (Merck) and a 95:5:0.1 mixture of dichloromethane, methanol, and 28% NH_3_ in H_2_O as the eluent to yield 0.4 g (64%) of 4-[(5-chloropyridin-2-yl)­methoxy]-1-[3-(propan-2-yl)-2,3,4,5-tetrahydro-1*H*-[1,4]­diazepino­[1,7-*a*]­indol-9-yl]­pyridin-2­(1*H*)-one (**37**) as a free base. HPLC-MS (ESI) Method 2 >99% 463.2 [M + H]^+^. Preparation of the maleic acid salt. To a solution of 4-[(5-chloropyridin-2-yl)­methoxy]-1-[3-(propan-2-yl)-2,3,4,5-tetrahydro-1*H*-[1,4]­diazepino­[1,7-*a*]­indol-9-yl]­pyridin-2­(1*H*)-one free base as described above (**37**, 0.47 g, 1.015 mmol) in a 10:1 mixture of dichloromethane and methanol, maleic acid (0.13 g, 1.117 mmol) was added, and the mixture was concentrated. The residue was triturated with ethanol, and after 1 h of stirring, the solid product was filtered, washed with ethanol, and dried to yield 0.57 g (96%) of 4-[(5-chloropyridin-2-yl)­methoxy]-1-[3-(propan-2-yl)-2,3,4,5-tetrahydro-1*H*-[1,4]­diazepino­[1,7-*a*]­indol-9-yl]­pyridin-2­(1*H*)-one (**37**) maleic acid salt. HPLC-MS (ESI) Method 2 >99% 463.2 [M + H]^+^. HPLC purity (Method 1): 99.28%. ^1^H NMR (500 MHz, DMSO-*d*_6_): δ 9.6 (br m, 1H, H-11), 8.67 (d, *J* = 2.5 Hz, 1H, H-28), 8.03 (dd, *J* = 8.4, 2.5 Hz, 1H, H-27), 7.61 (d, *J* = 8.4 Hz, 1H, H-25), 7.53–7.58 (m, 2H, H-5, H-16), 7.44 (d, *J* = 2.0 Hz, 1H, H-2), 7.05 (dd, *J* = 8.7, 2.0 Hz, 1H, H-6), 6.43 (s, 1H, H-7), 6.12 (dd, *J* = 7.6, 2.8 Hz, 1H, H-18), 6.05 (s, 2H, maleic acid –HC=CH−), 5.94 (d, *J* = 2.8 Hz, 1H, H-19), 5.23 (s, 2H, H_2_-22), 4.10–5.10 (br m, 2H, H_2_-13), 3.73 (br m, 1H, H-31), 2.70–4.00 (br m, 6H, H_2_-9, H_2_-12, H_2_-14), 1.26 (br d, *J* = 6.6 Hz, 6H, H3-32, H3-33). ^13^C NMR (DMSO-*d*_6_, 126 MHz): δ 167.0 [HOC=O maleic acid], 166.4 [C-20], 162.7 [C-24], 154.1 [C-23], 147.7 [C-28], 140.3 [C-8], 139.9 [C-16], 136.8 [C-27], 135.6 [C-3], 135.3 [−C= maleic acid], 132.9 [C-4], 130.4 [C-29], 127.0 [C-1], 123.4 [C-25], 119.8 [C-6], 118.0 [C-2], 109.4 [C-5], 100.4 [C-7], 99.5 [C-18], 97.9 [C-19], 69.7 [C-22], 59.2 [C-31], 51.5 [C-14], 49.8 [C-12], 40.4 [C-13], 24.3 [C-9], 16.4 [C-32, C-33]. ESI-HRMS: calcd for C_26_H_28_O_2_N_4_Cl [M + H]^+^, 463.18953; found, 463.18939; Δ = −0.3 ppm. ESI-HRMS: calcd for C_4_H_3_O_4_ [M – H]^−^, 115.00368; found, 115.00364; Δ = −0.4 ppm.

### Pharmacology

All animal experiments performed in this study were conducted in compliance with institutional guidelines. All studies and procedures were carried out on live animals at Richter Gedeon Plc. and complied with Act XXVIII of 1998 law on the Protection and Welfare of Animals as well as Hungarian Government Decree 40/2013 (II.14) on animal testing that is based on Directive 2010/63/EU on the protection of animals used for scientific purposes. The Decree ensures that no project is carried out without prior authorization of the national competent authority, the National Food Chain Safety Office. Projects may be carried out if the National Scientific Ethical Committee on Animal Experimentation gives a favorable project evaluation. The animal-welfare body of Gedeon Richter Plc. (Local Ethical Committee) preapproved the experiments of the project and reviewed the internal operational processes. Project License Numbers of studies listed in the article are the following: 22.1/1900/3/2011 (research and development of pharmaceutical agents for the treatment of obesity, eating disorders, and diseases affecting the metabolic system), 22.1/1902/3/2011 (general and safety pharmacology), and 22.1/1903/3/2011 (DMPK).

### Evaluation of MCHR1 Antagonism in a Functional Assay

Measuring of cytoplasmic calcium concentration ([Ca^2+^]_i_): cells (human MCH1 (SLC1) AequoScreen Cell Line, PerkinElmer ES-370-A, lot no. M4W-A2) expressing human MCH1 receptor, Aequorint and Gα16 were cultured in F12 medium (Gibco 21765) containing 10% FBS (Gibco 10500), 1× antibiotic antimycotic solution (Sigma A5955), 400 μg/mL G418 (Gibco 11811-023), and 250 μg/mL zeocin (Life Technologies R250-01).

One day before the [Ca^2+^]_i_ measurement, cells were plated in a 96-well plate (96-well plate, Costar 3595) at a density of 30,000 cells/well in the above-described culturing medium but without G418 and zeocin. On the day of measurement, the culture medium was removed from the cells, and a fluorescent Ca^2+^-sensitive dye (FLIPR calcium 5 kit, Molecular Devices R8186) was added to the cells at a 4× dilution compared to the recommended dilution by the manufacturer, in a volume of 100 μL/well, and cells were incubated at 37 °C for 10 min. DMSO stock solutions were made from the test compounds, which were diluted with an assay buffer (HEPES buffered salt solution (HBSS): 140 mM NaCl, 5 mM KCl, 10 mM HEPES, 2 mM CaCl_2_, 2 mM MgCl_2_, 20 mM glucose, pH 7.4, 305–315 mOsm + 2 mM probenecid (Sigma P8761)) (final DMSO concentration was 1%). The vehicle (DMSO, control treatment) or the buffer containing test compounds was added to the cells in a volume of 50 μL/well, and cells were incubated at 37 °C for further 60 min.

[Ca^2+^]_i_ measurement was carried out by a FlexStation II (Molecular Devices) plate reader fluorimeter (excitation 485 nm, emission 525 nm). MCH was used as the agonist (Bachem H-1482). A 1 mM stock solution was made from the agonist in distilled water, this solution was distributed into aliquots, which were kept at −20 °C until use. One aliquot was used only once. Fluorescence was detected before addition of MCH for 20 s, and after addition of MCH for 40 s. MCH was applied at an EC_80_ concentration; the EC_80_-values were determined individually for every plate/experiment. For this whole MCH dose–response curves were determined on one part of the plate, 4 parameter sigmoidal curves were fitted to the experimental data by nonlinear regression, MCH EC_80_ values were derived from the fitted curve. The raw fluorescence data were converted into Δ*F*/*F* values (the maximum fluorescence value obtained after addition of MCH was normalized to the baseline fluorescence: Δ*F*/*F* = (*F*_max_ – *F*_baseline_)/*F*_baseline_). The inhibitory potency of test compounds was expressed as percent inhibition calculated according to the following formula: inhibition % = 100 × (1 – (Δ*F*/*F*_compound_ – Δ*F*/F_DMSO buffer_)/(Δ*F*/*F*_MCH control_ – Δ*F*/*F*_DMSO buffer_)).

IC_50_ values of the tested compounds were determined by fitting 4 parameter sigmoidal curves to inhibition % data. Data processing, including fitting curves by nonlinear regression, was done with SoftMaxPro software.

### Intrinsic Clearance Assay

In vitro metabolic stability was assessed using human (Xenotech, LLC, USA), Wistar rat, and NMRI mouse (In vitro Metabolism Research, Gedeon Richter Plc, Hungary) liver microsomes. Test compounds were incubated at a 1 or 2.5 μM initial test concentration at the longest for up to 40 min with the liver microsomes (0.5 mg/mL). In vitro intrinsic clearance (CL_int_, μL/min/mg protein) was calculated by using the basic concept of clearance prediction according to the following equations: CL_int_ = *V*_max_/*K*_M_, or if *S* ≪ *K*_M_, CL_int_ = *V*/*S*; *V*_max_ = maximal rate of enzyme reaction; *K*_M_ = affinity constant of substrate concentration; *V* = actual rate of enzyme reaction under first order conditions, *S* = substrate concentration in the incubations.

### Permeability Assay

Bidirectional permeability (PappA-B and PappB-A) and efflux ratio (PDR = PappB-A/PappA-B) of test compounds were measured using vinblastine-treated Caco-2 (VB-Caco-2) cells described in ref 22. The permeability of test compounds (at 10 μM) was measured in the apical-to-basolateral (A–B) and basolateral-to-apical (B–A) directions in HBSS–HEPES (Hank’s buffered salt solution containing 25 mM HEPES) using isopH conditions (pH 7.4 A–7.4 B) at 37 °C with moderate shaking (120 rpm). The incubations with test compounds were performed at the longest up to 180 min, using appropriate duration of time determined based on preliminary studies for each compound tested. Samples were analyzed using an HPLC or UHPLC–MS/MS.

### Plasma Protein and Brain Tissue Binding Assay

The plasma protein and brain tissue binding of the test compound was measured at a test concentration of 3 μM in pooled fresh mouse plasma samples and brain homogenates using an equilibrium dialysis method. The samples were incubated for 5.5 h in the equilibrium dialysis apparatus in a shaking water bath maintained at 37 °C, and the postdialysis samples were analyzed by LC–MS/MS.

### Ex Vivo Receptor Occupancy Assay

The ex vivo receptor occupancy assay was performed using the radioligand [^3^H]­SNAP-7941 and mouse striatum homogenate.

Tritiated SNAP-7941 was synthesized following the method described in the literature, with minor modifications.^21^

Screen compounds were dissolved in the vehicle (i.e., 5% Tween-80/1% lactic acid in distilled water). Male mice (22 to 27 g) were treated intraperitoneally or gavaged with the vehicle (10 mL/kg) or screen compounds in the vehicle. Two hours after dosing, mice were decapitated and striatum was removed and frozen on dry ice. The bilateral striatum (40 mg/brain) was homogenized with 9-times volume of buffer and used immediately (4.4 mg wet tissue/well). Striatum homogenates were incubated with 0.3 nM [^3^H]­SNAP-7941 for 45 min at room temperature. The final incubation volume was 0.3 mL. Nonspecific binding, assessed in the presence of GW803430 (10 μM), was <10% of the total binding. The assay was stopped by filtration on a Unifilter GF/C glass fiber filter presoaked for 2–3 h in a 0.5% polyethylene–imine solution before use. The filters were washed 3 times with 1 mL of assay buffer and radioactivity retained in the filters was determined with a TopCount liquid scintillation counter. Ex vivo receptor labeling by [^3^H]­SNAP-7941 in drug-treated animals was calculated and expressed as follows: receptor occupancy (%) = 100 × [1 – (receptor labeling of drug-treated group/receptor labeling of vehicle-treated group)].

### Diet-Induced Obesity (DIO) Model and Assay

Group-housed C57BL/6 mice weighing ∼22–25 g were kept on a reverse day–night cycle (light off: 12 h, lights on: 24 h) and fed with a 60% fat-containing diet until the body weight reached 45 g on average (12 weeks fatting). Following isolation and 4 days of per os treatment with tap water (habituation), mice were administered (po) once (between 10 and 12 h) or twice (between 10 and 12 h and 16–18 h) daily with test compounds or the vehicle for 14 days (first day of treatment: D-0). Body weight and chow consumed were measured daily before the first daily treatment. Obese mice were fed with a high-fat-containing diet (Research Diets D12492) and lean mice with a low-fat-containing diet (Research Diets D12450B) during the whole experiment. Doses were calculated in terms of base. Control-subtracted body weight loss data were analyzed with one -way ANOVA and a Tukey HSD post hoc test using Statistica 10.1 of Statsoft Inc.

### Pharmacokinetics

The brain penetrability of **37** was investigated after oral administration of the compound in 5% Tween 80 in deionized water to male NMRI mice at a dose of 10 mg/kg (10 mL/kg). Animals (*n* = 5/sampling time) were exsanguinated at 30 min, 1 h, 2 h, 5 h, 24 h, and 48 h after treatment and whole brains were also removed. Blood samples were taken in Li-heparin tubes and centrifuged at 2000*g* for 20 min at 4 °C to gain plasma. Brain homogenates were prepared by homogenizing the whole brain tissues with deionized water (brain/water 1:2.5 w/w). After sample preparation (extraction with chlorobutane after alkalization with 28% NH_3_ in H_2_O) plasma and brain homogenate samples were analyzed for the test compound concentration by HPLC-UV technique. An Agilent 1200 HPLC instrument with stationary phase Kinetex, XB-C18 (50 × 3 mm, 2.6 μm), and mobile phase of methanol and 0.1 M NH_4_OAc was used for analysis. Gradient elution was used with a flow rate of 0.5 mL/min. Detection wavelength was set at 234 nm. Pharmacokinetic parameters were calculated from the mean time–concentration curves by Kinetica 4.4.1 software (Thermo Fisher scientific), using the model-independent approach. The brain penetrability was estimated from the brain/plasma ratio calculated from the AUC (area under the curve) values up to the last measured brain or plasma concentration.

Plasma and brain concentrations of **37** were measured in a mouse Diet-Induced Obesity (DIO) test. Blood and whole brain samples were prepared as given above. The gained plasma and brain homogenate samples were processed by protein precipitation with acetonitrile and measured for **37** concentrations by the HPLC–MS/MS technique. An Agilent 1200 HPLC system with an AB Sciex QTRAP 4500 mass spectrometer was used for the analysis. The separation was performed on a Kinetex XB C18 (50 × 2.1 mm, 2.6 μm) column with eluent 0.1% formic acid in deionized water and 0.1% formic acid in acetonitrile. The flow rate was set at 600 μL/min and gradient elution was used. Detection was at mass transitions at *m*/*z* 463.2 → 421.1 Da.

### Electrophysiology

The patch clamp assay for the measurement of hERG currents was performed by B’SYS GmbH (Switzerland).^36^

The 293 cell line stably expressing the transcripts of human K_v_11.1 (hERG) was generated by bSys GmbH (Switzerland). The cells were cultured 1:1 mixture of Dulbecco’s modified Eagle medium and nutrient mixture F-12 (D-MEM/F-12 1×, liquid, with l-glutamine) supplemented with 10% FBS and 1.0% penicillin/streptomycin solution, 0.1 mg/mL hygromycin B, and 15 μg/mL blasticidin. Cultures were kept at 37 °C in 5% CO_2_. For electrophysiological measurements, cells were seeded onto sterile culture dishes containing 2 mL of culture medium without antibiotics.

Whole-cell patch clamp recordings were performed manually (EPC-9, HEKA Electronics; Patchmaster). The culture dishes upon which cells were seeded at a density allowing single cells to be recorded were placed on the dish holder of the microscope and continuously perfused (at approximately 1 mL/min) with the bath solution (137 mM NaCl, 4 mM KCl, 10 mM HEPES, 1.8 mM CaCl_2_, 1 mM MgCl_2_, 10 mM glucose, and 0.3% DMSO; pH 7.4). Recording patch pipettes were filled with the intracellular solution (130 mM KCl, 5 mM EGTA, 10 mM HEPES, 1 mM MgCl_2_, and 5 mM ATP. pH 7.2). Measurements were performed at 36 ± 1 °C.

hERG outward tail currents were measured upon depolarization of the cell membrane to +20 mV for 2 s (activation of channels) from a holding potential of −80 mV and upon subsequent repolarization to −40 mV for 3 s. This voltage protocol was run at least 15 times at intervals of 10 s. If the current amplitude was judged to be too low (<500 pA) for measurement, another cell was recorded. Once control recordings have been accomplished (less than 5% change of current amplitude within 150 s), cells were continuously perfused with a test solution or 0.3% DMSO.

The test solutions of **37** were prepared by diluting the 333× DMSO containing stock solutions with a bath solution. Control experiments were performed by treating the cells with 0.3% DMSO. Peak amplitudes of the tail current evoked by the −40 mV step potential were measured from the baseline current (i.e., the current value before the prepulse command). The percentage inhibition was calculated from the comparison of peak currents in the presence and absence of the test compound. These values of relative current block were run-down corrected by normalizing the residual values obtained with 0.3% DMSO treated cells. Pooled data are presented as the mean ± SEM. The dose–response curve was generated, and the IC_50_ and Hill coefficient were calculated using a SigmaPlot 11.0. For the determination of the IC_50_ value, the test item concentrations found during the dose formulation analysis of the samples taken after experimentation were used.

### Modeling

Schrödinger software package (Schrödinger Release 2024-4: Prime; Induced Fit Docking; Glide; Maestro; Desmond; FEP+ Schrödinger, LLC, New York, NY, 2024) was used for molecular modeling and visualization.

Primarily the recently published experimental structure of MCHR1 (PDB id: 8WSS)^25^ was used for modeling protein–ligand interactions which was compared to the inactive state model of MCHR1 (version 2024-05-15) downloaded from the GPCRdb Web site.^37^ The structures were prepared using Schrödinger’s LigPrep protocol with default settings. The prepared experimental structure was analyzed with SiteMap^38^ and compound **37** was docked with induced-fit protocol^25^ to the assumed binding site leading to at most 20 refined protein ligand complexes by default. Two poses (poses 1 and 2) were selected for further refinement with 200 ns molecular dynamics (MD) simulations with Desmond. The system was surrounded by explicit cell membrane components and water molecules in a periodic system. The system was allowed to relax and sample the conformational space, resulting in a much more complex picture than a simple docked model. In these simulations, only the chain R was used and harmonic force constant of 1 kcal/mol/Å^2^ was applied for all Cα atoms in transmembrane helices to preserve the main protein conformation. To position the cell membrane model (POPC) the transformed protein structure available in the PDBTM database was used.^39^ Other parameters are summarized in Table S1 in the Supporting Information. The range 100–200 ns of trajectory belonging to pose 1 was analyzed using the simulation interaction diagram tool.

The MD refined protein–ligand complex was used as the initial structure for FEP calculations with Schrödinger’s FEP+ protocol.^33–35^ Compounds **1**, **16**, **30,** and **36** were aligned to **37** based on maximum common substructure, and we used the “optimal” protocol for generating FEP map, which was manually adjusted. For more details, see Tables S2–S5.

## Supplementary Material







## Notes

Spectroscopic data, analytical HPLC trace of compounds, and modeling (PDF)
3D models used for FEP+ calculations as initial structures (ZIP)
Molecular formula strings (CSV)

## Abbreviations

BWL: body weight loss
FEP: free-energy perturbation
IFD: induced-fit docking
LH: lateral hypothalamic
MCH1, MCHR1: melanin-concentrating hormone receptor 1
MD: molecular dynamics
NHP: nonhuman primate
ORX: orexinergic
POPC: 1-palmitoyl-2-oleoyl-*sn*-glycero-3-phosphocholine
PWS: Prader–Willi syndrome
SGF: simulated gastric fluid
SIF: simulated intestinal fluid
SNORD116: small nucleolar RNA, C/D box 116
TdP: torsades de pointes
