# Supplementary material for: The Discovery of RGH-706, a Highly Efficacious MCH1 Receptor Antagonist, for the Treatment of Obesity and Insatiable Hunger
Source: J Med Chem. 2026 Jan 22;69(8):8722–38. doi: 10.1021/acs.jmedchem.5c02708 (PMC13126673; doi:10.1021/acs.jmedchem.5c02708)
Supplement: Supplementary file 1 [file jm5c02708_si_001.pdf]

Supporting Information

The Discovery of RGH-706, a Highly Efficacious MCH1 Receptor Antagonist for the Treatment of Obesity and Insatiable Hunger

Gyula Beke,*[1] András Boros,[1] György M. Keserű,[2] Balázs Krámos,[1] Krisztina Katalin Szalai,[1] Anikó Gere,[1] Mónika Vastag,[1] Márta Thán,[1] Balázs Varga,[3] Ottilia Balázs,[1] Sándor Farkas,[4] Balázs Lendvai[1], István Greiner,[1] János Éles*[1]

[1]Gedeon Richter Plc., 19-21 Gyömrői út, Budapest 1103, Hungary

[2]Medicinal Chemistry Research Group, Research Centre for Natural Sciences, Budapest 1117, Hungary

[3]Department of Neurosurgery, Stanford School of Medicine, MSLS Building, 1201 Welch Rd, Stanford, CA 94305, USA

[4]VRG Therapeutics, 3 Füvészkert utca, Budapest 1083, Hungary

Corresponding author information

*(Gy.B.) Phone: +36-208298919; Email: bekegy@gedeonrichter.com

*(J.É.) Phone: +36-202731325; Email: elesj@gedeonrichter.com

Table of contents

S1 Title page

S2 NMR spectra and HPLC analitical traces for target compounds with *in vitro*/*in vivo* data

S15 Modeling

HPLC analitical trace for compound 7

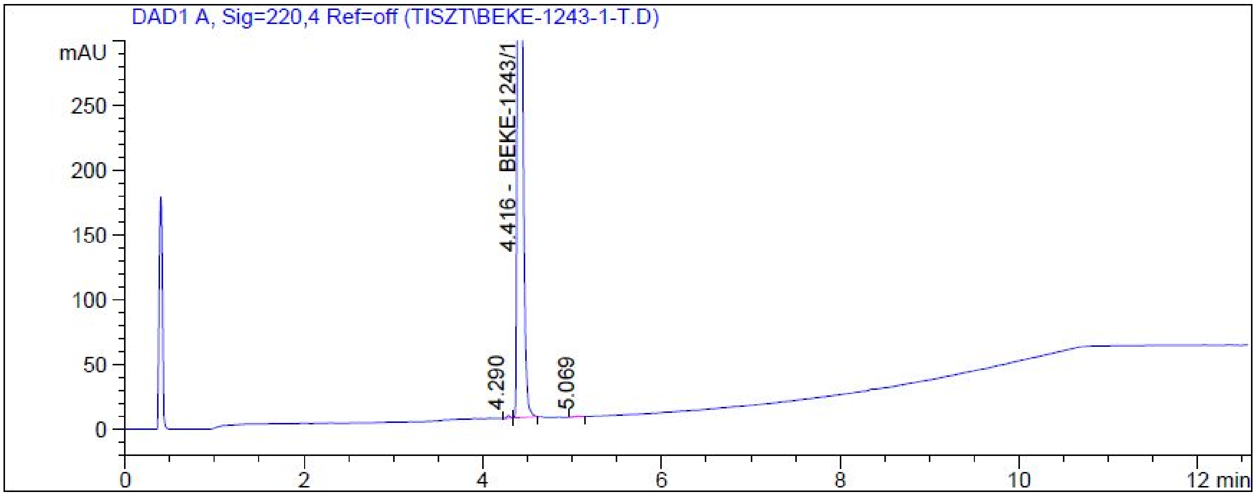

#	Meas. Ret. Time	Height	Area	Area %
1	4.290	2.390	6.753	0.22
2	4.416	964.864	3107.038	99.69
3	5.069	0.561	2.923	0.09

NMR spectra for compound 7

^{1}H (CD_2Cl_2, 500 MHz)

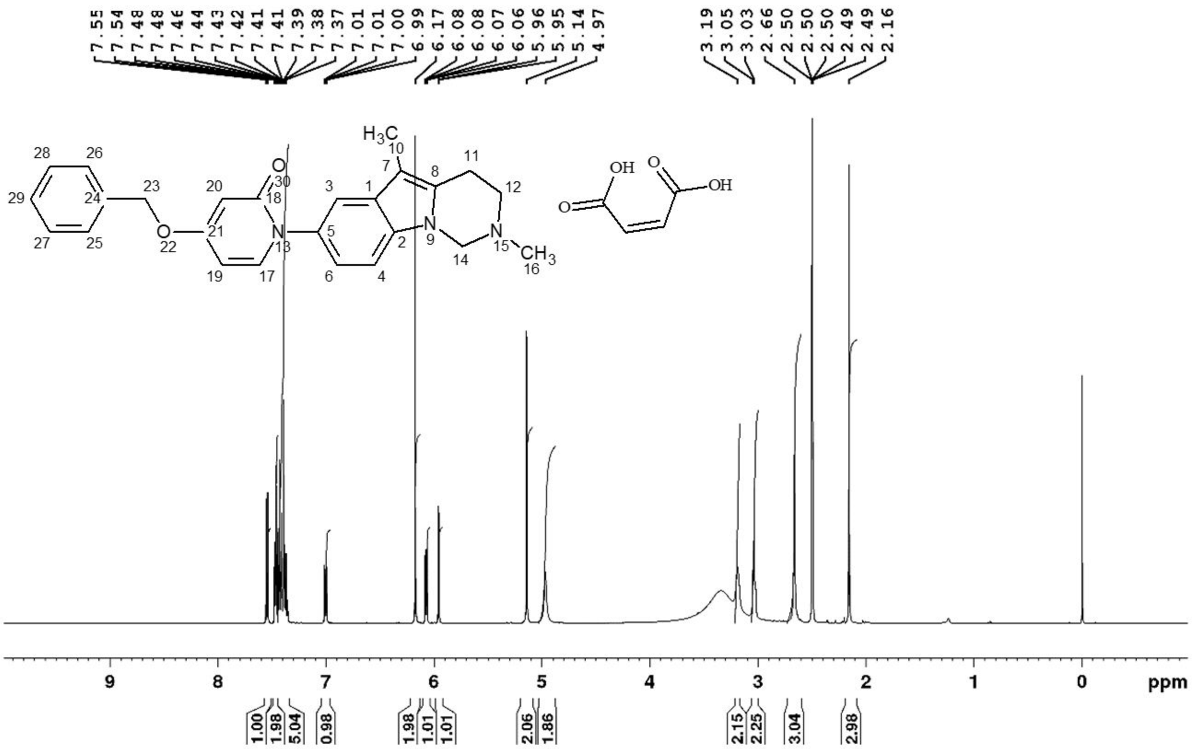

$^{13}C\{^{1}H\}$ (CD_2Cl_2, 500 MHz)

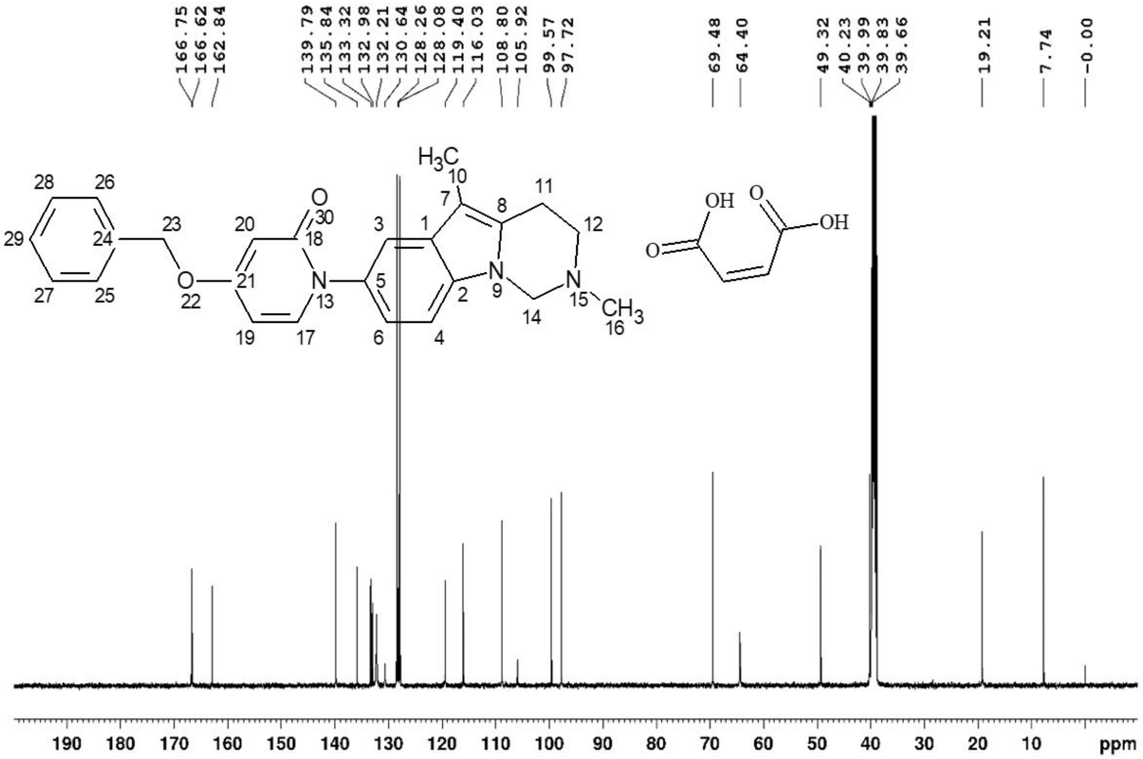

HPLC analitical trace for compound 16

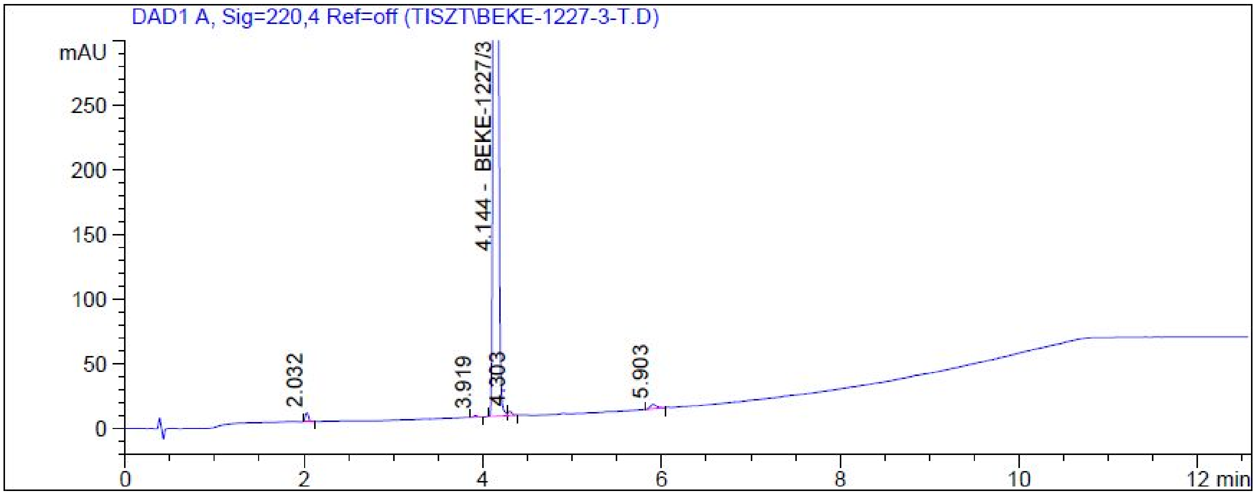

#	Meas. Ret. Time	Height	Area	Area %
1	2.032	6.881	17.524	0.43
2	3.919	1.343	3.559	0.09
3	4.144	1326.088	3991.361	98.82
4	4.303	3.093	10.330	0.26
5	5.903	3.515	16.278	0.40

NMR spectra for compound 16

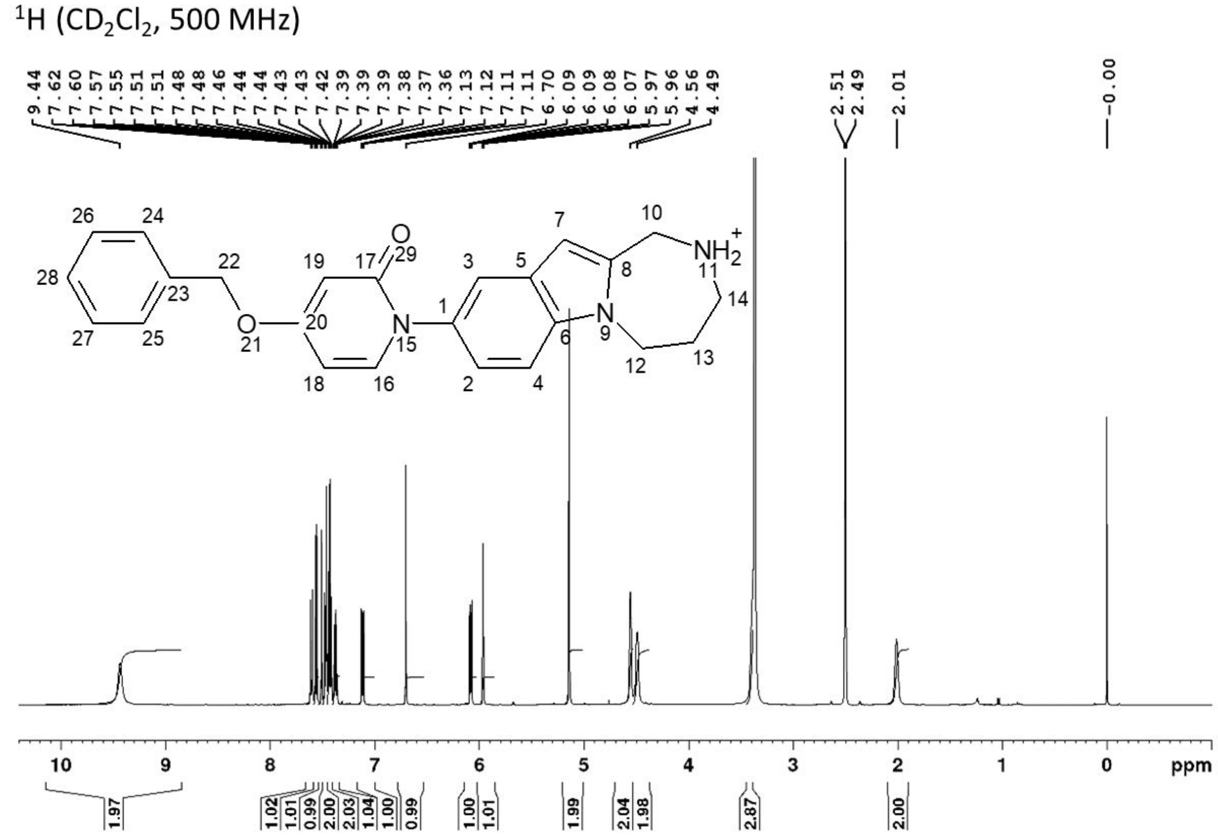

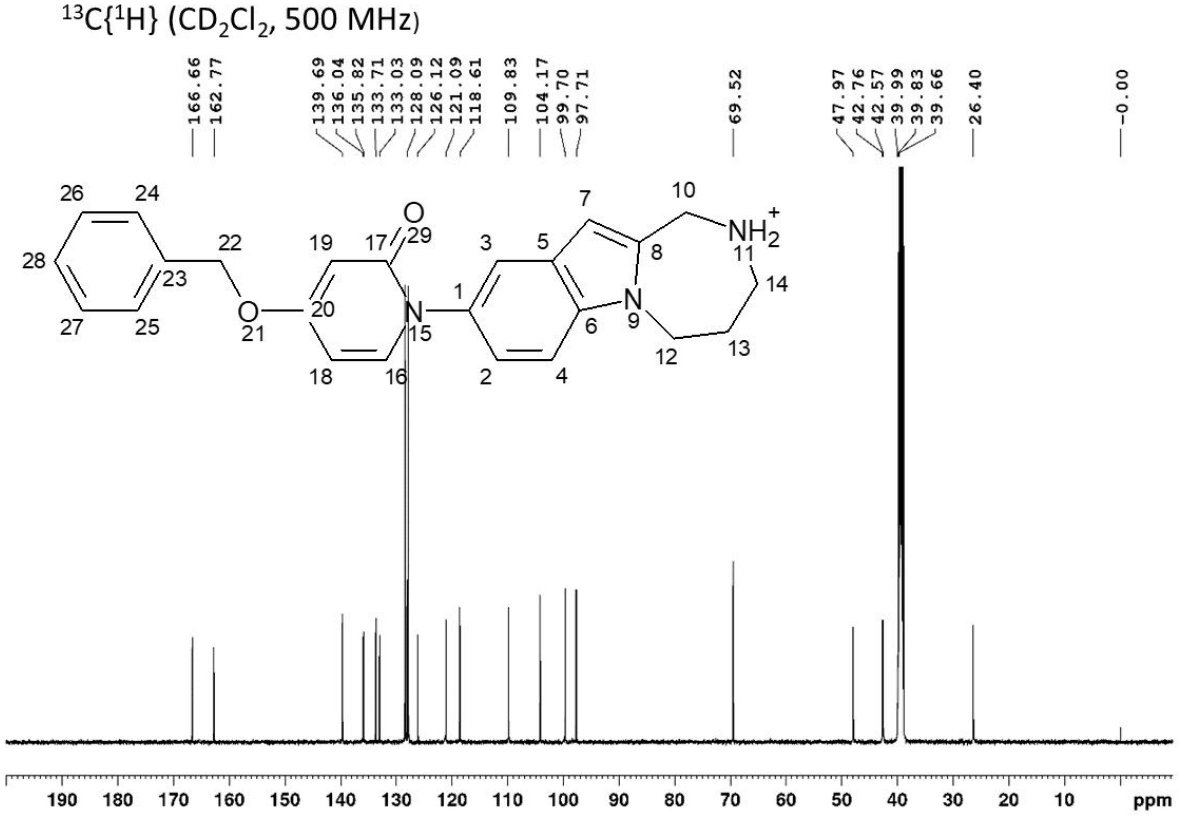

HPLC analitical trace for compound 30

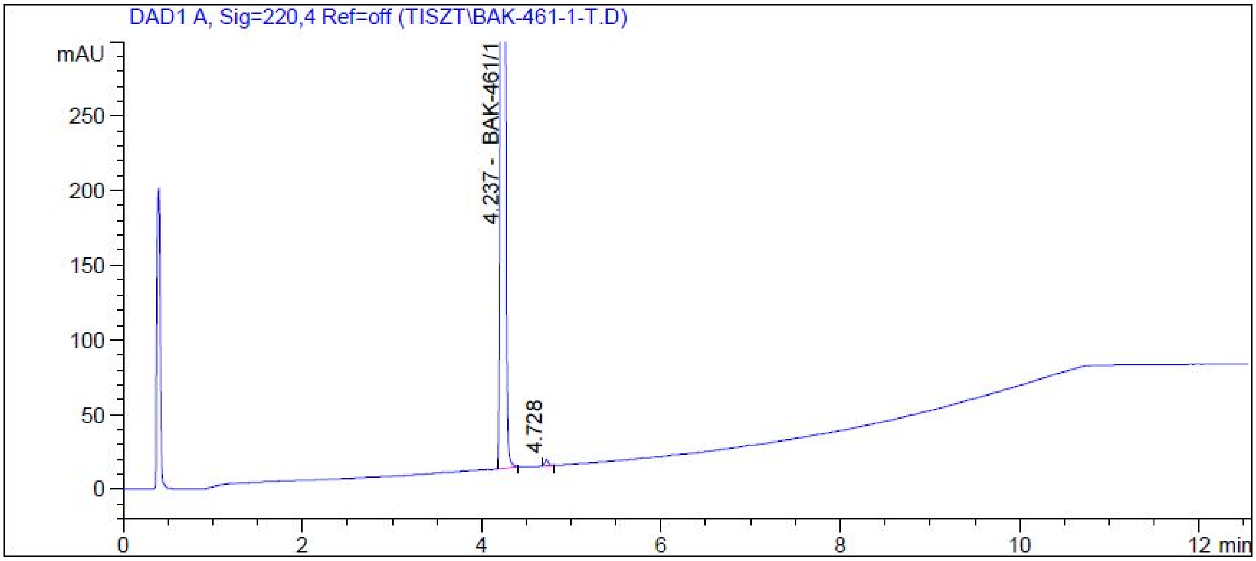

#	Meas. Ret. Time	Height	Area	Area %
1	4.237	1196.280	3613.286	99.66
2	4.728	4.157	12.252	0.34

NMR spectra for compound 30

^{1}H (DMSO-d_6, 500 MHz)

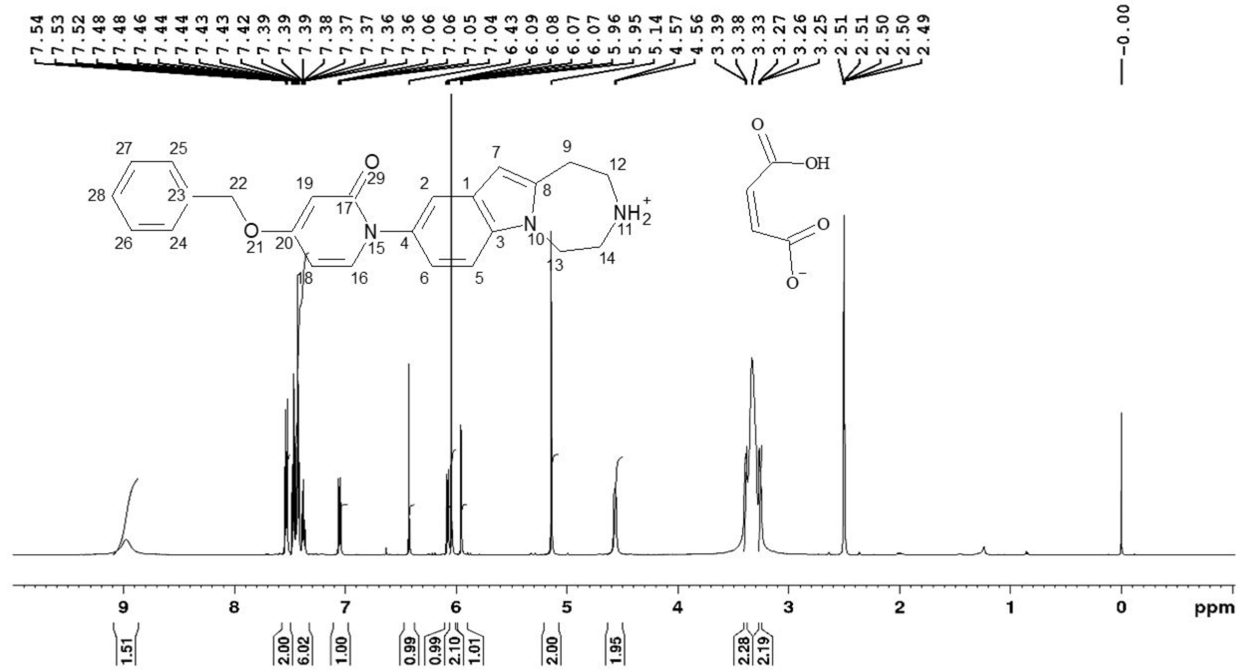

$^{13}C\{^1H\}$ (DMSO-d_6, 500 MHz)

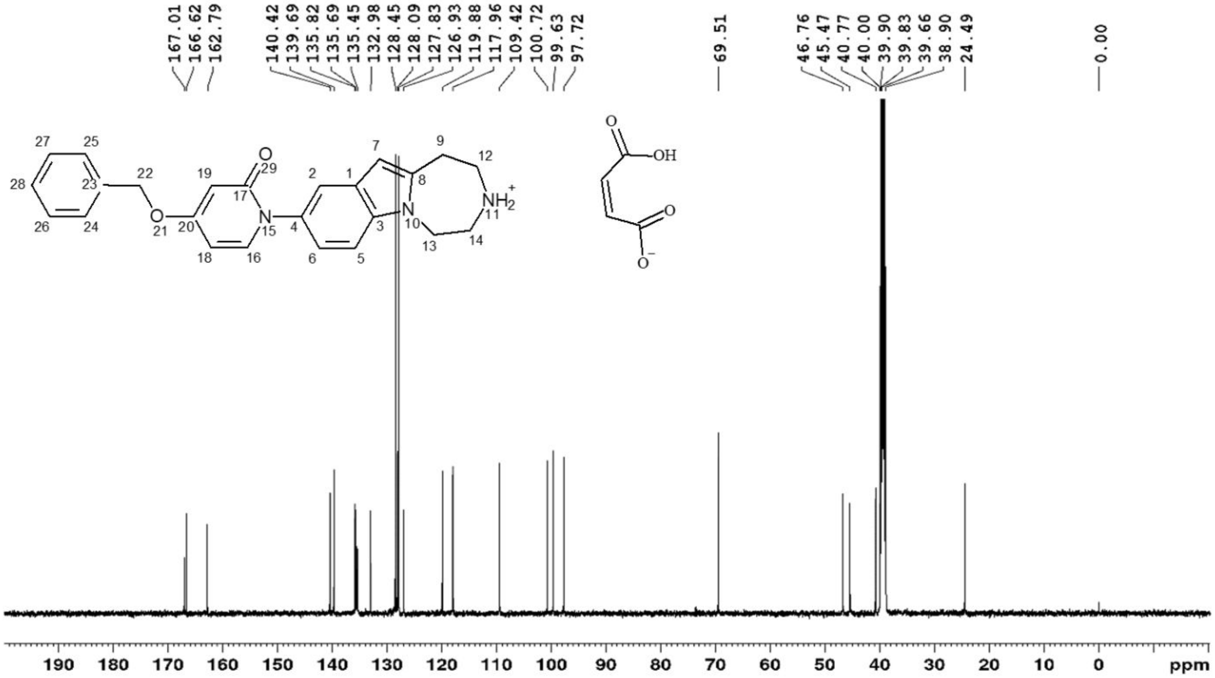

HPLC analitical trace for compound 36

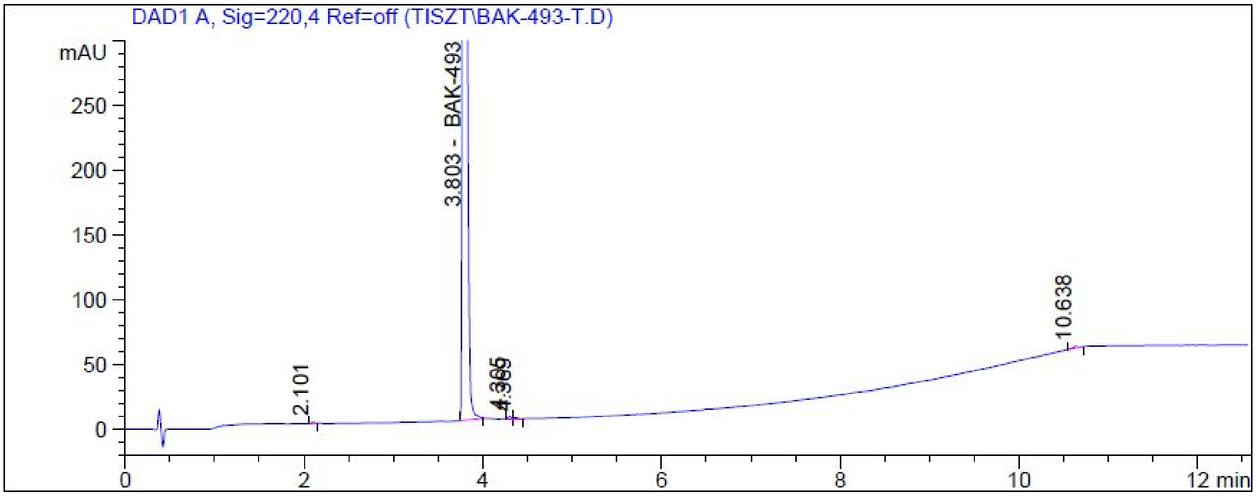

#	Meas. Ret. Time	Height	Area	Area %
1	2.101	0.829	2.080	0.06
2	3.803	1145.853	3375.375	99.50
3	4.305	2.142	5.844	0.17
4	4.369	1.037	2.787	0.08
5	10.638	1.465	6.237	0.18

NMR spectra for compound 36

^1H (DMSO-d_6, 500 MHz)

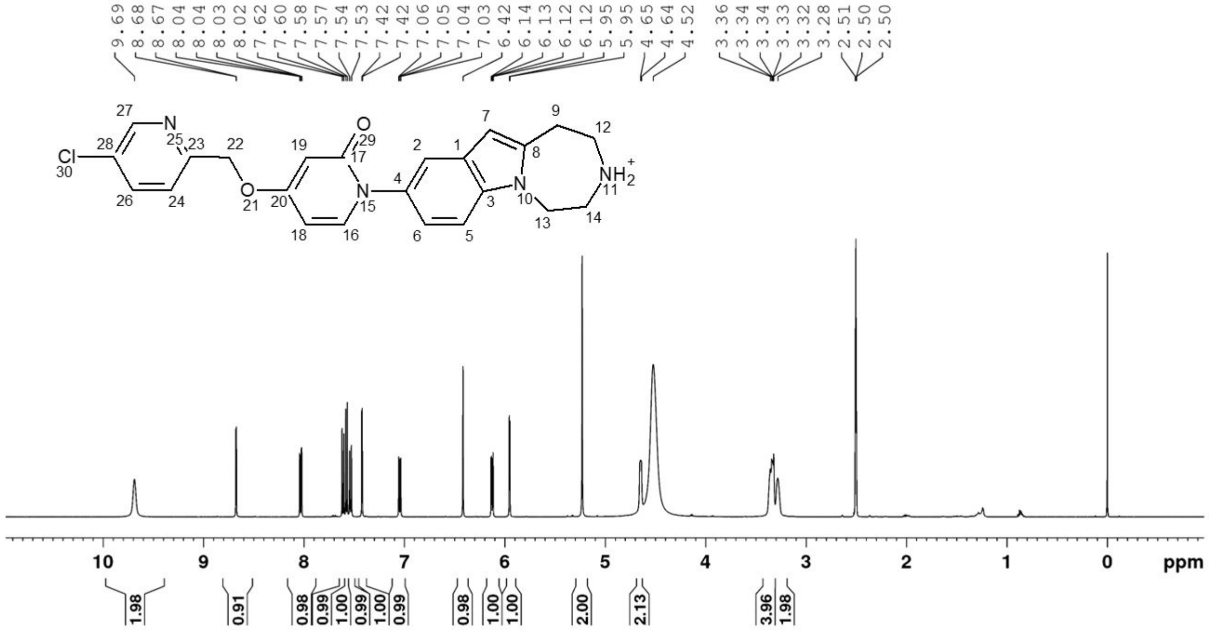

$^{13}C\{^1H\}$ (DMSO-d_6, 500 MHz)

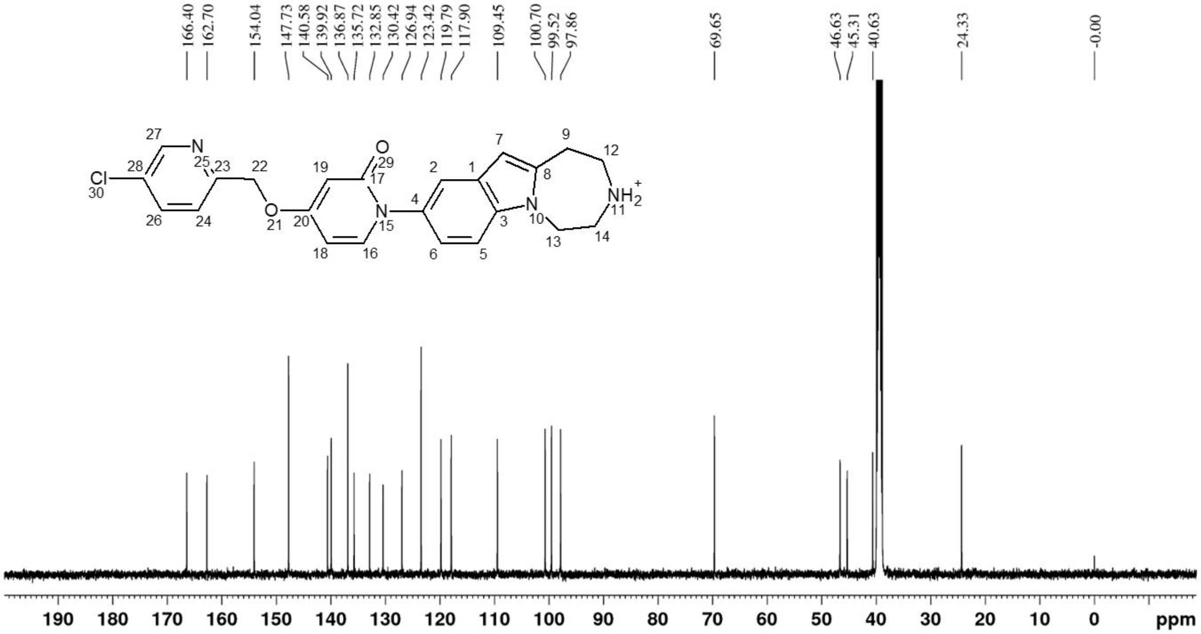

HPLC analitical trace for compound 37

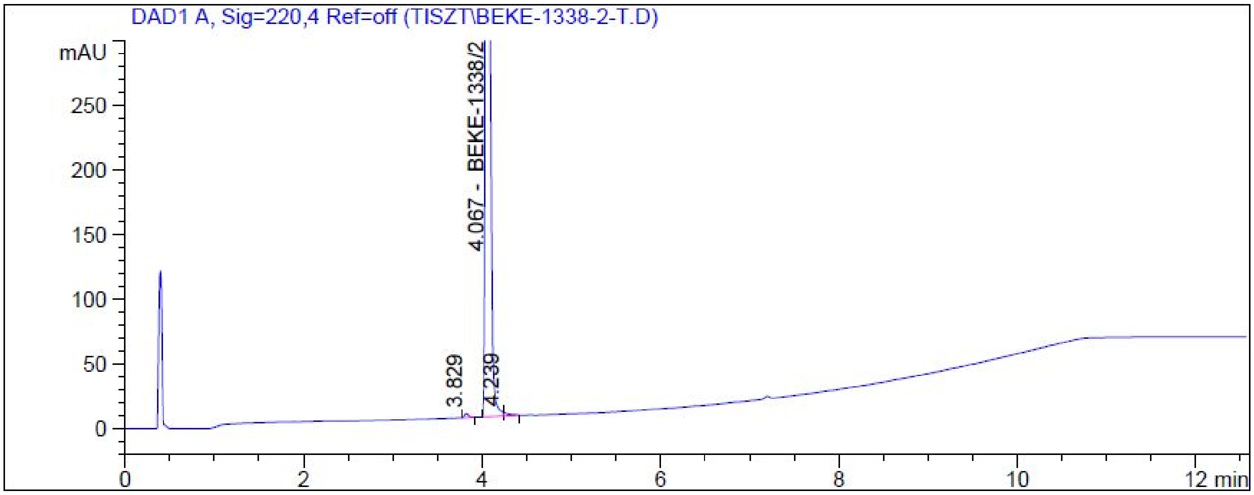

#	Meas. Ret. Time	Height	Area	Area %
1	3.829	3.109	9.064	0.34
2	4.067	860.614	2651.396	99.28
3	4.239	2.847	10.252	0.38

NMR spectra for compound 37

^1H (DMSO-d_6, 500 MHz)

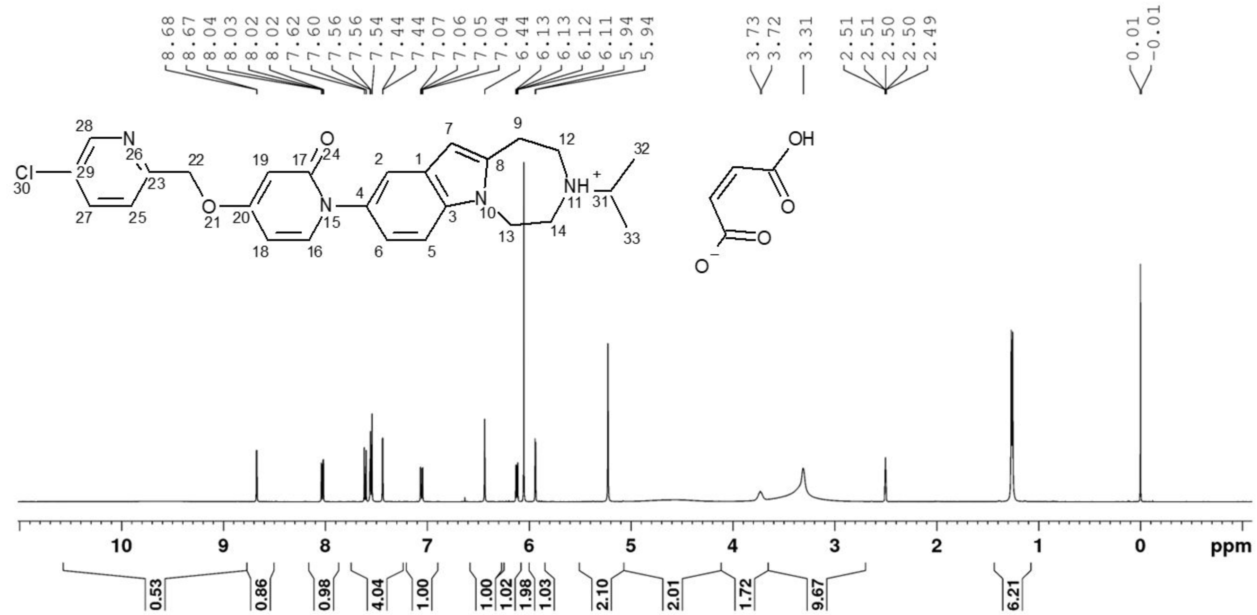

$^{13}C\{^1H\}$ (DMSO-d_6, 500 MHz)

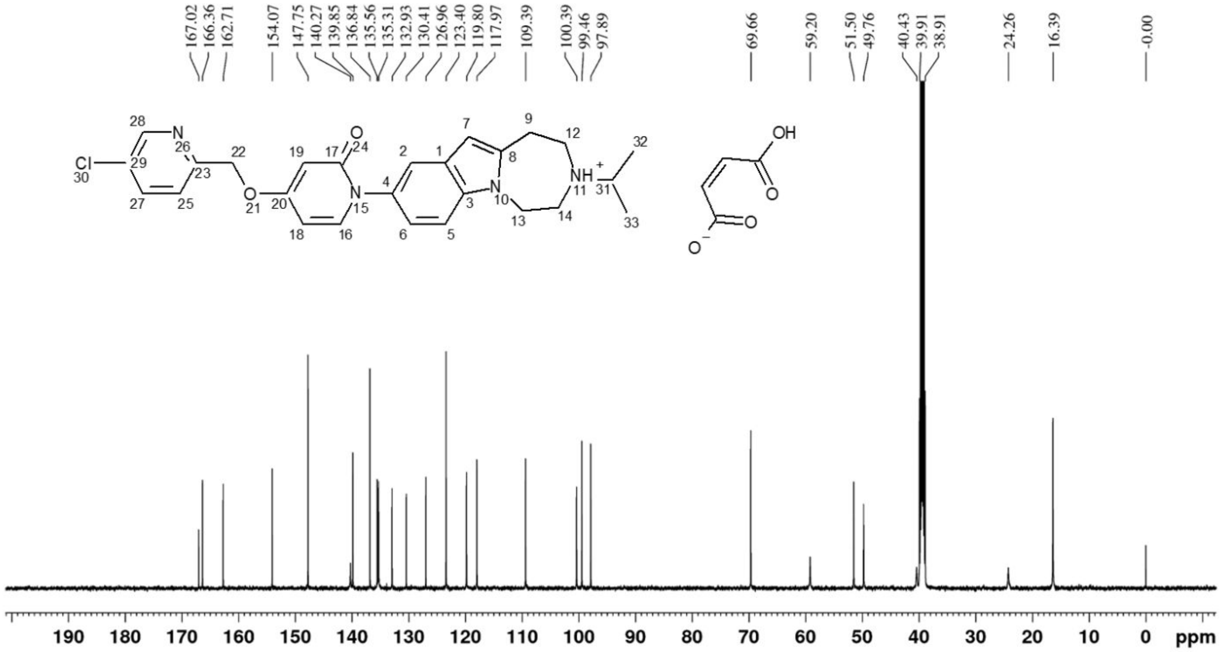

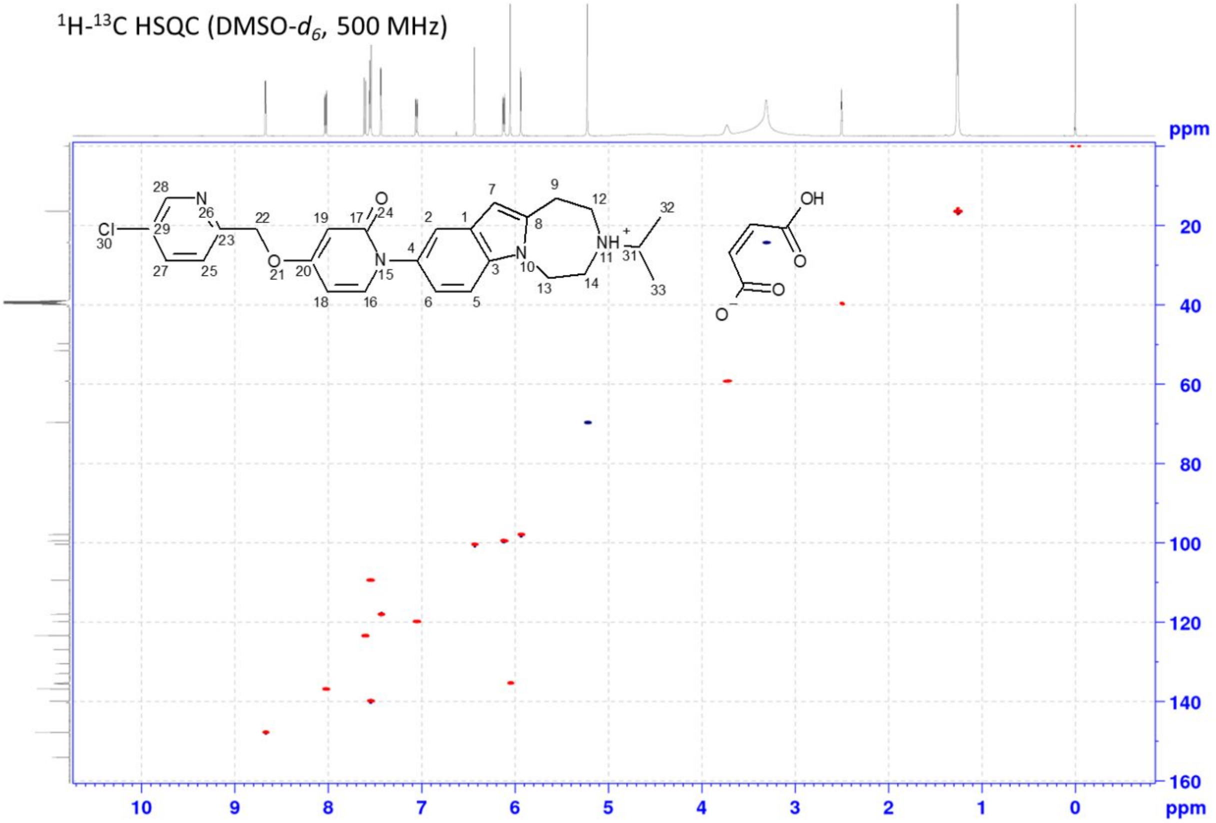
^{1}H-^{13}C HSQC (DMSO-d_6, 500 MHz)
ppm
ppm

^{1}H-^{13}C HMBC (DMSO-d_6, 500 MHz)

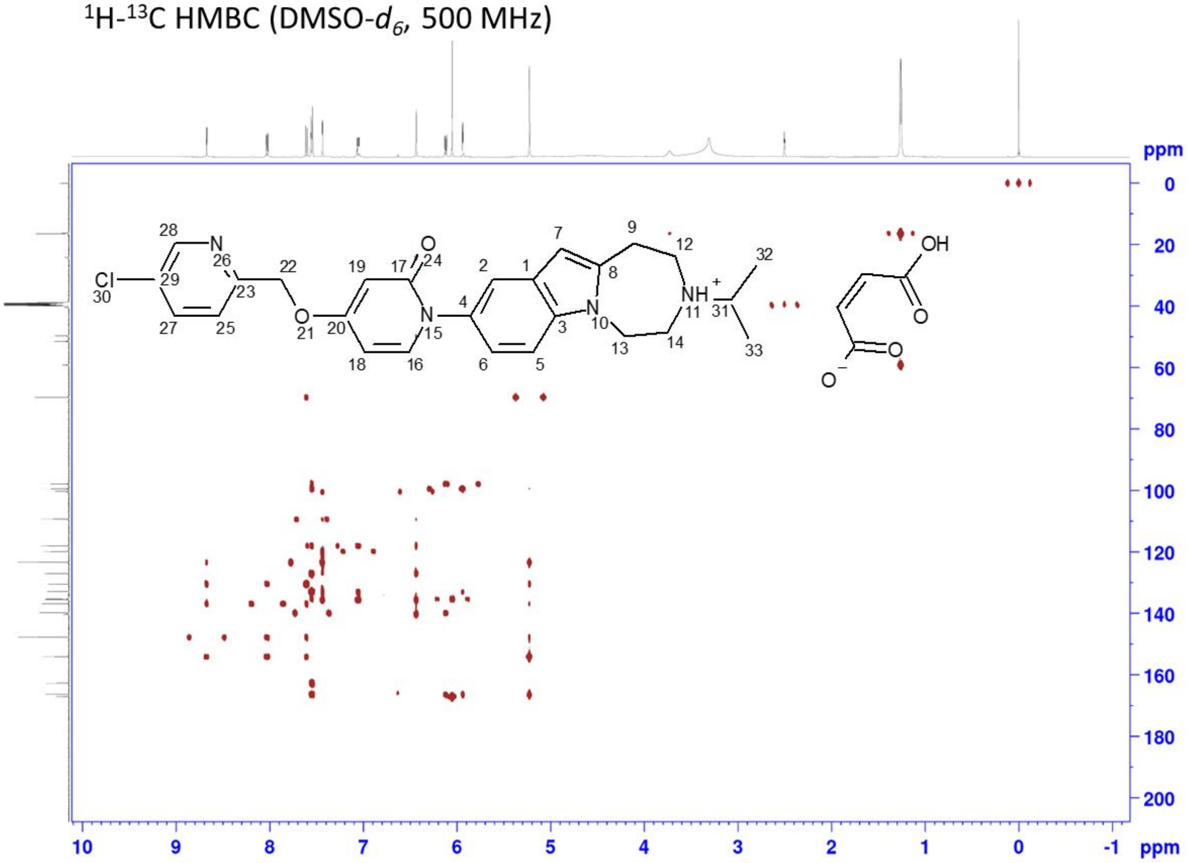

^{1}H-^{1}H NOESY (DMSO-d_6, 500 MHz)

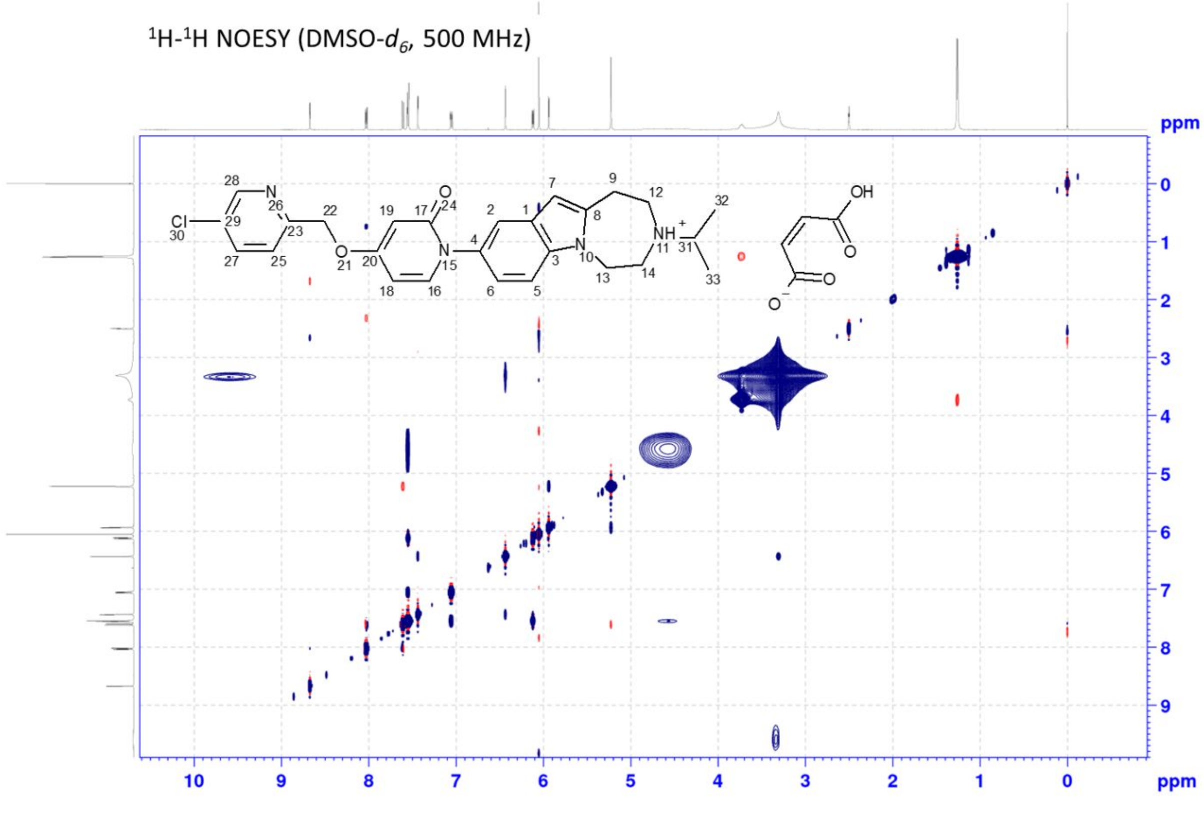

^{1}H-^{1}H COSY (DMSO-d_6, 500 MHz)

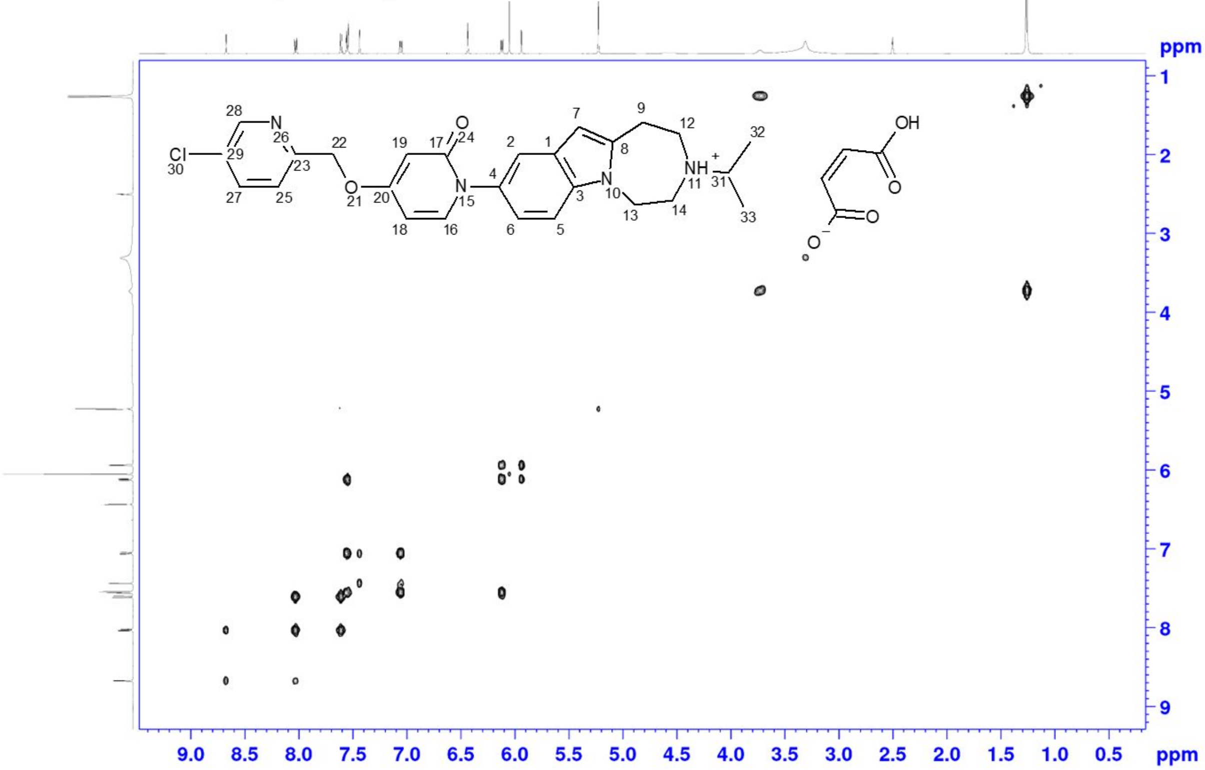

^{1}H-^{1}H TOCSY (DMSO-d_6, 500 MHz)

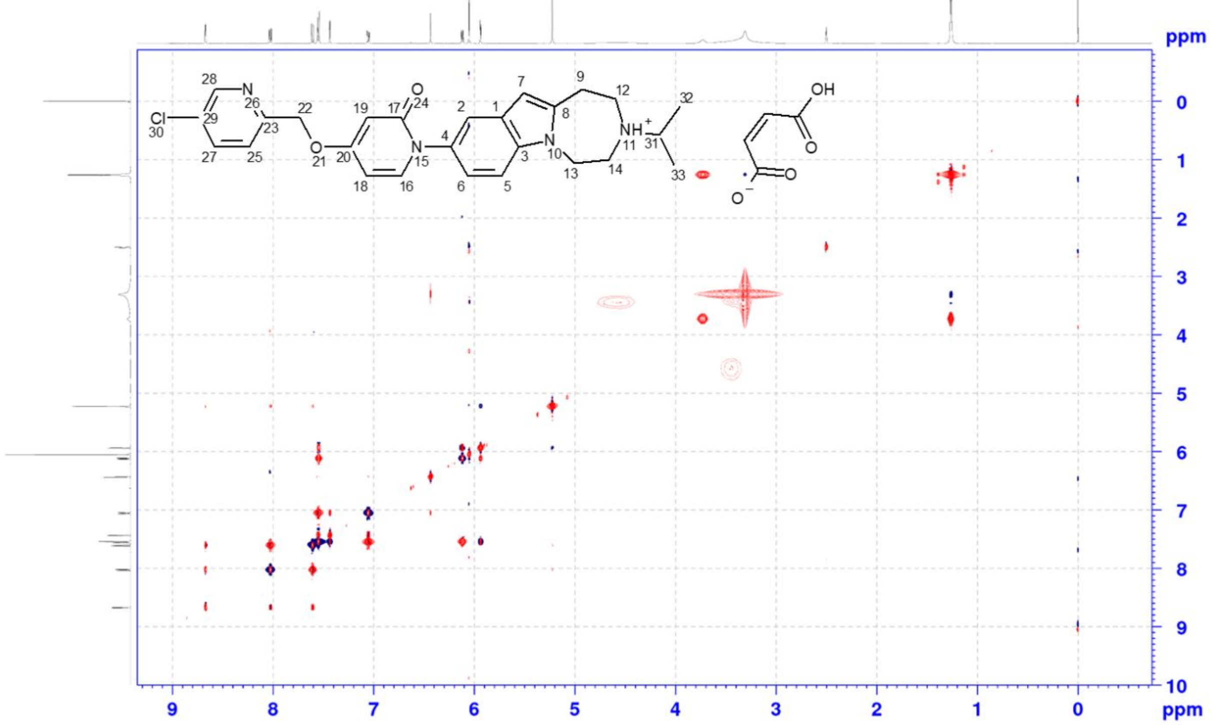

Modeling

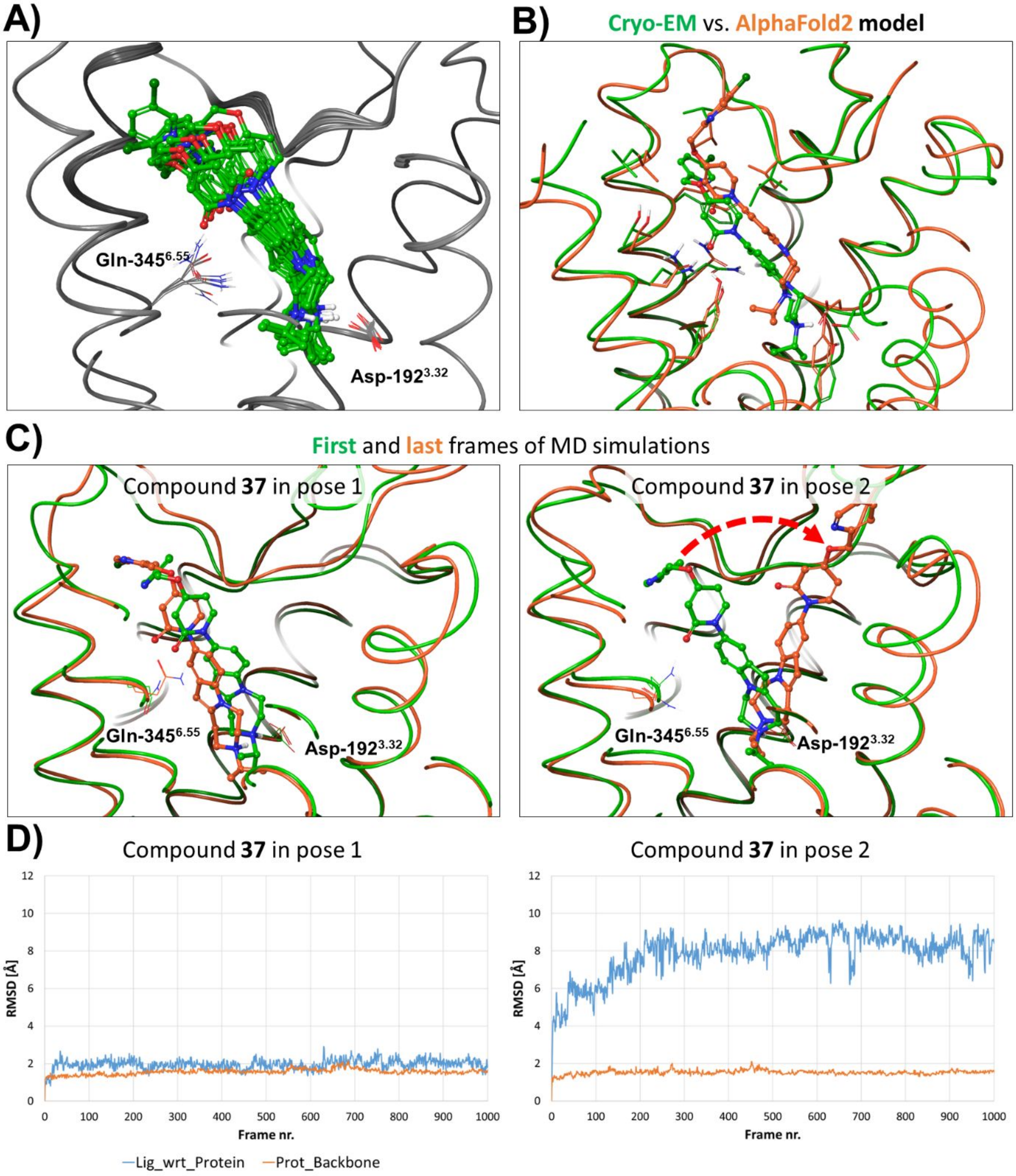

Figure S1. Supporting figures: A) Top 10 binding poses of compound **37** suggested by induced-fit dockings starting from the cryo-EM structure (PDB id: 8WSS); B) AlphaFold2 model aligned

to the cryo-EM structure. Compound **37** was docked with induced-fit protocol (pose 1 is presented); C) Comparison of the first and the last frame of MD simulations where compound **37** was in pose 1 and pose 2 at the binding site and D) the corresponding RMSD curves. Pose 1 was suggested to be stable by these calculations.

Since the experimental structure used for modeling belongs to an MCH-activated receptor and the compounds we studied were antagonists, we examined how this state differs from the inactive conformation available from the GPCRdb database1, to estimate the extent of rearrangement expected at the binding site during receptor activation. The comparison showed that the shape of the protein backbone and the binding site is similar, but the small differences highly influenced how the ligands could be docked (Figure S1/B), and the binding pose seemed to be better in the cryo-EM structure, supporting the application of the experimental structure.

FEP edge: 1 >> 16

Difference in binding free energy (ΔΔG) is: 1.31 kcal/mol

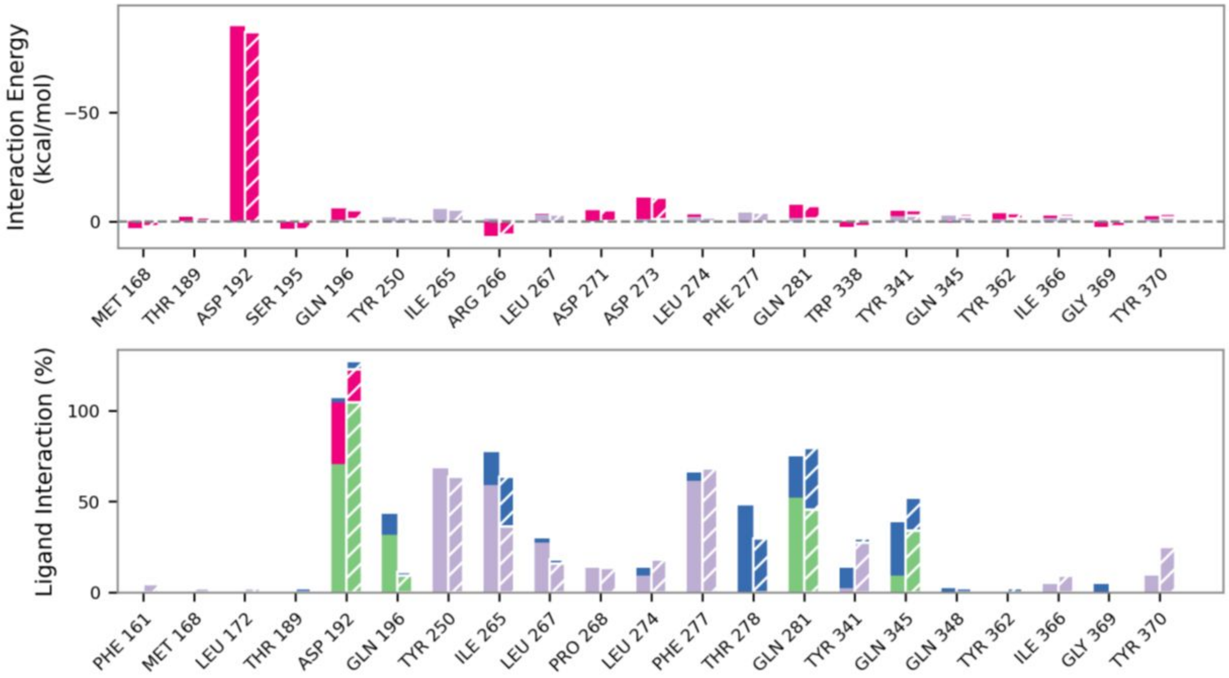

FEP edge: 30 >> 16

Difference in binding free energy (ΔΔG) is: 1.89 kcal/mol

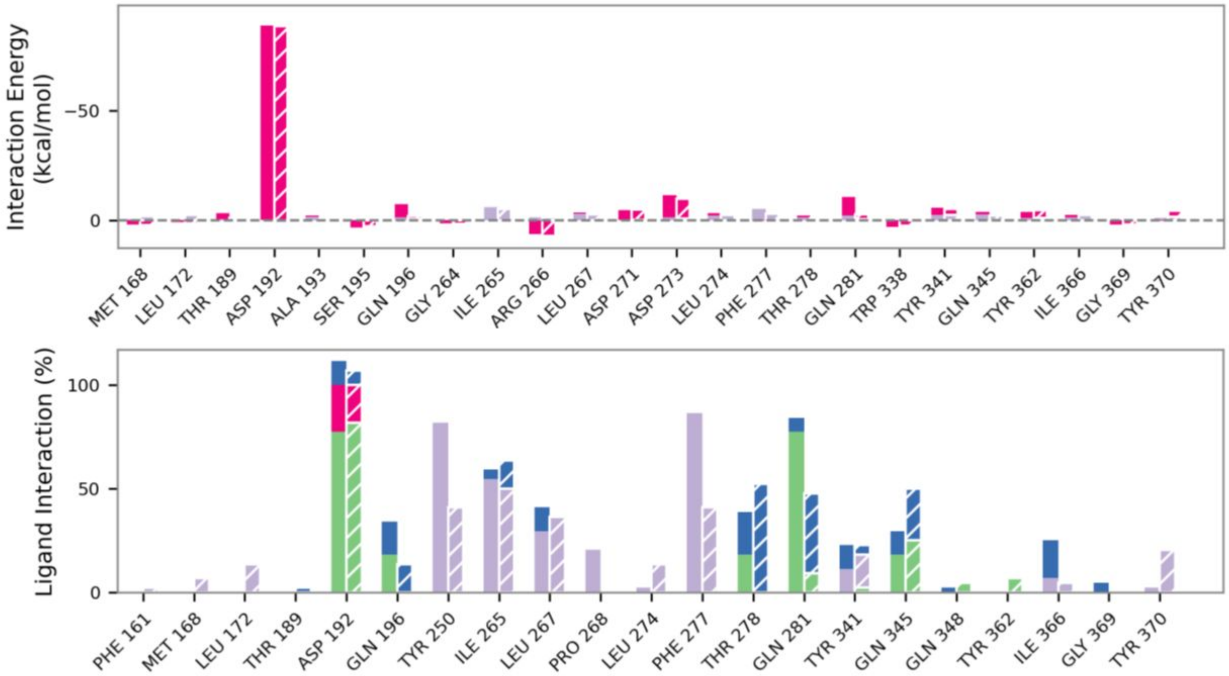

Figure S2. Protein-ligand interactions based on highlighted edges from FEP+ simulation: Upper charts illustrate receptor's per-residue interaction energy. Solid bars show interactions with Ligand 1, while dashed bars with Ligand 2. The energy is decomposed into electrostatics and Van der Waals terms. For clarity, interactions with an absolute total energy less than 2.0 kcal/mol are excluded; Negative energies are attractive, positive are repulsive interactions.

Lower charts illustrate the types of interactions receptor residues have with ligands and their frequencies throughout the simulation. Solid bars show interactions with Ligand 1, while dashed bars with Ligand 2. Specific interactions are tracked throughout the simulation, offering insights into how the receptor engages with each ligand. These differences might explain changes in binding energy between the two ligands. The interactions monitored and displayed include hydrogen bond, halogen bond, hydrophobic, ionic and water bridge. The stacked bar charts are normalized over the course of the trajectory. Below are the same interactions projected on a ligands' 2D-structure. More details about the geometry and interaction categories can be found in the Schrödinger's Desmond User Manual, under 'Simulation Interactions Diagram' (SID) section. The values may exceed 100% as the residue can make multiple contacts of the same type with the ligand; For clarity, all interactions that are less than 20% are excluded. Based on the analysis, the overall calculated free energy differences cannot be solely attributed to the change of a single interaction. This is because, during thermal motions, the compounds invariably strive to form the salt bridge, which represents the most significant interaction. This, in turn, leads to changes in the other interactions as well.

Table S1. MD refinement settings

Phase	Parameter	Value
system building	solvent model	TIP3P
	membrane model	POPC (300K)<br>it was positioned using structure from the PDBTM database
	box shape	orthorombic
	box size	buffer (10x10x10 Å)
	force field	OPLS4 (with custom parameters generated automatically by the force field builder module)
	ions	32 Cl^- and 18 Na^+ (neutralization and additional salt in 0.15 M concentration)
MD minimization	simulation time	100 ps
	restraints	harmonic force constant of 1 kcal/mol/Å^2 was applied for all Cα in transmembrane helices
MD simulation	relaxation method	default system relaxation protocol
	simulation time	200 ns
	ensemble class	NPγT
	temperature	300 K
	pressure	1.01325 bar
	surface tension	0.0 bar·Å
	recording interval	200 ps for trajectory
		10 ps for energy
	number of frames	1000
	integration (RESPA)	bonded: 2.0 fs, near: 2.0 fs far: 6.0 fs
	thermostat	Nose-Hoover chain; relaxation time: 1.0 ps

	barostat	Martyna-Tobias-Klein; relaxation time: 1.0 ps; coupling style: isotropic
	Coulombic interaction cutoff	9 Å
	restraints	same as in MD minimization
	seed	2007
MD minimization (leading to initial structure for FEP calculations)	simulation time	100 ps
	restraints	harmonic force constant of 1 kcal/mol/Å^2 was applied for all Cα in transmembrane helices

Table S2. Frequency of specific protein-ligand interactions in MD simulation; Different types of interactions are counted in the range of 100 ns to 200 ns part of the MD trajectory (500 frame), where compound **37** was in pose 1. In this table the ratio of the MD frames is summarized in which the geometric criteria of a given interaction type met. It should be noted that a single residue can form more than one counted interaction with the ligand at the same time, so the sum can be higher than 100%.

Residue	Salt bridge	Hydrogen bond	Water bridge	π-π	Cation-Pi	Other hydrophobic	Halogen bond	Sum
Phe-161	-	-	-	-	-	5.6%	-	5.6%
Asp-192	90.7%	2.8%	63.0%	-	-	-	-	156.5%
Ala-193	-	-	-	-	-	1.4%	-	1.4%
Tyr-250	-	-	-	3.8%	-	2.8%	-	6.6%
Ile-265	-	-	8.9%	-	-	49.3%	-	58.3%

Leu-267	-	-	2.2%	-	-	30.8%	-	33.0%
Pro-268	-	-	-	-	-	5.2%	-	5.2%
Thr-272	-	-	-	-	-	-	0.4%	0.4%
Leu-274	-	-	4.4%	-	-	16.5%	-	20.9%
Phe-277	-	-	0.2%	20.9%	-	43.7%	-	64.8%
Thr-278	-	-	27.6%	-	-	-	-	27.6%
Gln-281	-	98.6%	4.4%	-	-	-	-	103.0%
Trp-338	-	-	-	-	-	0.6%	-	0.6%
Tyr-341	-	-	1.2%	17.3%	41.4%	31.4%	-	91.3%
Gln-345	-	12.7%	6.6%	-	-	-	-	19.3%
Gln-348	-	0.2%	1.6%	-	-	-	-	1.8%
Tyr-362	-	-	1.0%	-	-	-	-	1.0%
Ile-366	-	-	-	-	-	4.0%	-	4.0%
Tyr-370	-	-	-	-	72.4%	7.2%	-	79.5%

Table S3. FEP+ settings

Parameter	Value
map	initial map was generated using the “optimal” option, which was manually completed (Table SX4)
force field	OPLS5 (with custom parameters generated automatically by the force field builder module)
solvent model	SPC
ensemble class	μVT
simulation time	10.0 ns

number of λ-windows	for default perturbation protocol: 12<br>for core-hopping perturbation protocol: 16<br>for charge-hopping: 24
system builder buffer size	5.0 Å
custom charges mode	assign
calculate relative solvation	yes
add salt	no
restraints	no
seed	2014

Table S4. Calculated FEP edges (alchemical transformations) and predicted free energy differences

Edge<br>ligand1 >> ligand2	Protocol	Pred. ΔΔG [kcal/mol] (raw)	Pred. ΔΔG [kcal/mol] (corrected)
1 >> 16	core-hopping	1.31±0.13	1.31±0.13
16 >> 37	charge0	-1.83±0.22	-2.63±1.39
30 >> 16	charge0	1.89±0.12	2.08±0.88
30 >> 37	default	-0.72±0.23	-0.55±1.16
36 >> 16	charge0	2.9±0.17	1.92±1.39
36 >> 30	default	-0.53±0.18	-0.16±1.16
36 >> 37	default	-1.33±0.2	-0.71±1.39

Table S5. Calculated relative binding free energies (FEP results)

Compound	Pred. ΔΔG [kcal/mol] (raw)
**1**	-1.3±0.42
**16**	0±0.4
**30**	-2.1±0.97
**36**	-1.9±1.44
**37**	-2.6±1.44

Reference

1. Pándy-Szekeres, G.; Caroli, J.; Mamyrbekov, A.; Kermani, A. A.; Keserű, Gy. M.; Kooistra, A. J.; Gloriam, D. E. GPCRdb in 2023: state-specific structure models using AlphaFold2 and new ligand resources. Nucleic Acids Res. [Online] 2023, 51, D395–D402. https://academic.oup.com/nar/article/51/D1/D395/6827106 (accessed September 2, 2025).
